# miR482f and miR482c-5p from edible plant-derived foods inhibit the expression of pro-inflammatory genes in human THP-1 macrophages

**DOI:** 10.3389/fnut.2023.1287312

**Published:** 2023-11-30

**Authors:** Ester Díez-Sainz, Silvia Lorente-Cebrián, Paula Aranaz, Ez-Zoubir Amri, José I. Riezu-Boj, Fermín I. Milagro

**Affiliations:** ^1^Department of Nutrition, Food Science and Physiology/Center for Nutrition Research, Faculty of Pharmacy and Nutrition, University of Navarra, Pamplona, Spain; ^2^Department of Pharmacology, Physiology and Legal and Forensic Medicine, Faculty of Health and Sport Science, University of Zaragoza, Zaragoza, Spain; ^3^Instituto Agroalimentario de Aragón-IA2, Universidad de Zaragoza-CITA, Zaragoza, Spain; ^4^Aragón Health Research Institute (IIS-Aragon), Zaragoza, Spain; ^5^Navarra Institute for Health Research (IdiSNA), Pamplona, Spain; ^6^CNRS, Inserm, iBV, Université Côte d'Azur, Nice, France; ^7^Centro de Investigación Biomédica en Red Fisiopatología de la Obesidad y Nutrición (CIBERobn), Instituto de Salud Carlos III, Madrid, Spain

**Keywords:** *CLEC7A*, *NFAM1*, *TLR6*, microRNA, cross-kingdom regulation, diet, inflammation, xenomiRs

## Abstract

**Background:**

Edible plants can exert anti-inflammatory activities in humans, being potentially useful in the treatment of inflammatory diseases. Plant-derived microRNAs have emerged as cross-kingdom gene expression regulators and could act as bioactive molecules involved in the beneficial effects of some edible plants. We investigated the role of edible plant-derived microRNAs in the modulation of pro-inflammatory human genes.

**Methods:**

MicroRNAs from plant-derived foods were identified by next-generation sequencing. MicroRNAs with inflammatory putative targets were selected, after performing *in silico* analyses. The expression of candidate plant-derived miRNAs was analyzed by qPCR in edible plant-derived foods and their effects were evaluated in THP-1 monocytes differentiated to macrophages. The bioavailability of candidate plant miRNAs in humans was evaluated in feces and serum samples by qPCR.

**Results:**

miR482f and miR482c-5p are present in several edible plant-derived foods, such as fruits, vegetables, and cooked legumes and cereals, and fats and oils. Transfections with miR482f and miR482c-5p mimics decreased the gene expression of *CLEC7A* and *NFAM1*, and *TRL6*, respectively, in human THP-1 monocytes differentiated to macrophages, which had an impact on gene expression profile of inflammatory biomarkers. Both microRNAs (miR482f and miR482c-5p) resisted degradation during digestion and were detected in human feces, although not in serum.

**Conclusion:**

Our findings suggest that miR482f and miR482c-5p can promote an anti-inflammatory gene expression profile in human macrophages *in vitro* and their bioavailability in humans can be achieved through diet, but eventually restricted at the gut level.

## Graphical Abstract

Image designed with BioRender. 
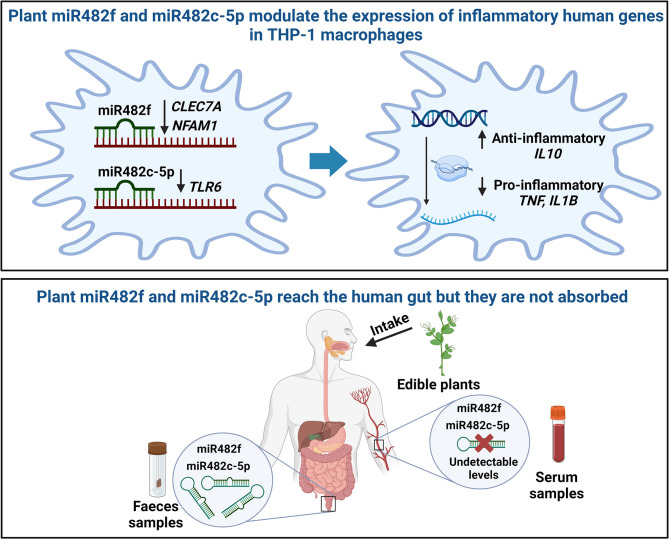

## 1 Introduction

Chronic inflammation is associated with the development of several comorbidities, such as cardiovascular diseases, cancer, autoimmune disorders, metabolic syndrome, type 2 diabetes, and non-alcoholic fatty liver disease, which altogether are the leading causes of mortality worldwide (1–4). Among the factors that could trigger long-term inflammation are obesity, chronic infections, gut microbiota dysbiosis, social, lifestyle, and environmental factors (1). The mechanisms underlying inflammation are complex and comprise several signaling pathways, including the nuclear factor kappa-B (NF-κB), mitogen-activated protein kinase (MAPK), and Janus kinase (JAK)-signal transducer and activator of transcription (STAT) pathways (5). Factors that could regulate these inflammatory pathways include Toll-like receptors (TLRs), microbial products, and pro-inflammatory cytokines, such as tumor necrosis factor-α (TNF-α) and interleukin-1β (IL-1β) (5).

Plant-based diets have been associated with the improvement of inflammation. Reported evidence has shown that consumption of nuts, fruits, vegetables, grains, and olive oil promotes an anti-inflammatory profile (6–11). However, the role of plant-based diets in inflammation in humans is currently under investigation; in particular, the identification of all bioactive molecules and their specific mechanisms of action. Among the plant bioactive compounds with anti-inflammatory activities are the phytochemicals, vitamins, minerals, and oil compounds, such as phenolics and triterpenoids found in fruits and vegetables; lectins and peptides in legumes; fiber, polyphenols, phytosterols, monounsaturated fatty acids (MUFAs), polyunsaturated fatty acids (PUFAs), vitamin E, selenium, and copper in nuts and peanuts; and polyphenols and fiber in cereals (6, 12–14). Notably, microRNAs (miRNAs) have recently emerged as new key bioactive molecules in plants (15, 16).

miRNAs are non-coding RNA molecules of approximately 22 nucleotides that exert post-transcriptional gene expression regulation by interacting with messenger RNAs (mRNAs) (17). Plant-derived miRNAs are crucial components of the biology of plants, playing a part in a wide range of functions, such as stress responses, development, and metabolism (18). Increasing evidence shows that edible plant-derived miRNAs could also modulate mammalian gene expression, influencing their physiology, for which they have become cross-kingdom gene expression regulators, and they stand for as therapeutic tools to treat human diseases (15, 16, 19, 20). Plant-derived miRNAs could modulate host metabolism, cell proliferation, apoptosis, and viral infections by interacting with diverse cell types, such as adipocytes, hepatocytes, enterocytes, and tumoral cells (21–27). However, there are controversial findings about the bioavailability of edible plant-derived miRNAs in the host (28). While some authors have found plant-derived miRNAs in animal tissues and blood samples, others have reported undetectable levels of these molecules in the host (29, 30). Further investigation is mandatory in order to clarify whether miRNAs could be relevant bioactive components of edible plants in modulating host physiology.

The general aim of the present study was to identify miRNAs in edible plant-based foods that could regulate the expression of human genes associated with inflammation. For this purpose, we selected several food types, including fruits, vegetables, legumes, cereals, and fats and oils, that have been previously associated with beneficial anti-inflammatory effects (6–11). The first objective consisted of identifying miRNAs in edible plant-based products that could display anti-inflammatory effects through modulation of human genes, by performing next-generation sequencing (NGS) and bioinformatic analysis to predict potential human target genes. The second objective was to evaluate the role of candidate edible plant-derived miRNAs in the modulation of inflammation-related human genes in human THP-1 macrophages. The third objective was to determine if candidate edible plant-derived miRNAs with potential anti-inflammatory effects could reach the gastrointestinal tract, being detected in human feces and serum samples.

## 2 Materials and methods

### 2.1 Total RNA isolation from samples of edible plant-derived foods

Plant fruits (apples, oranges, and pears), vegetables and greens (green peppers, spinaches, green beans, lettuces, and tomatoes), fats and oils (walnuts and olives), legumes (chickpeas and lentils), and cereals (rice) were purchased at local supermarkets. Plant-origin products were considered as different food types or groups: fruits (*n* = 3), vegetables/greens (*n* = 5), fats and oils (*n* = 2), and cooked legumes/cereals (*n* = 3). Green beans were heat treated in boiling water for 4.5 min in a pressure cooker. Legumes were soaked in water (lentils for 2 h and chickpeas for 12 h) and cooked in a pressure cooker (lentils for 15 min and chickpeas for 30 min). Rice was cooked in water for 20 min in a casserole. Other food elements were used as raw products (non-heat treated).

Total RNA was isolated using the miRNeasy Serum/Plasma Kit (Qiagen, Hilden, Germany) as follows: 0.1 g of fruits, legumes, vegetables, and greens, 0.05–0.1 g of fats and oils (0.05 g of walnut and 0.1 g of olive), and 0.2 g of cereals were added to 1 ml of QIAzol Lysis Reagent and grounded at the highest speed, on ice, using Ultra-Turrax T25 Basic (Basic IKA-Werke, Staufen, Germany) between 1 min (fruits, fats and oils, vegetables, and greens) and 2 min (legumes and cereals). Samples were centrifuged at 12,000 *g* between 5 min (fruits, walnuts, legumes, and cereals) and 15 min (olives, vegetables, and greens) at 4°C to remove cell debris and insoluble material. The upper-fat layer of olive samples was removed, and olive samples were centrifuged again at 12,000 *g* for 5 min at 4°C. The supernatants were collected, and RNA purification was concluded following the manufacturer's instructions. For the plant-based food samples used for quantitative real-time PCR (qPCR) assays, 1 μl of spike-in controls (RNA Spike-In Kit, for RT, Qiagen) was added to each sample. RNA for NGS or qPCR was isolated from one sample per type of plant-based food product.

### 2.2 Next-generation sequencing of miRNAs derived from samples of edible plant-based foods and bioinformatic analysis

NGS was performed in total RNA derived from plant-based products, which were selected for their suitability for small RNA-sequencing (small RNA-seq) after conducting quality controls: 0.1 g of fruits (apple, orange, and pear), 0.1 g of vegetables (spinach and tomato), and 0.05–0.1 g of fats and oil nuts (0.05 g of walnut and 0.1 g of olive). One sample was used for each type of plant-based food product. Libraries were prepared using the NEBNext^®^ Small RNA Library Prep Set for Illumina^®^ kit (New England Biolabs, Ipswich, MA, USA) according to the manufacturer's protocol. Briefly, RNAs were subjected to 3′ and 5′ adaptor ligation and first-strand cDNA synthesis. PCR selectively enriched those DNA fragments that had adapter molecules on both ends. Library amplification was performed by PCR using NEBNext^®^ Multiplex Oligos for Illumina (Index Primers Set 1–4) (New England Biolabs). Purification after PCR was performed using AgenCourt AMPure XP beads (Beckman Coulter, Brea, CA, USA), and libraries were analyzed using Agilent Bioanalyzer (Agilent Technologies, Santa Clara, CA, USA) to estimate the quantity and check size distribution. The samples underwent bead-based purification to remove big fragments: the samples were incubated with AgenCourt AMPure XP beads at a ratio of 1.3×, and the beads were then discarded. New beads were added to the supernatant for a final ratio of 3.7×, and all material left was recovered. Final libraries were quantified by qPCR using the KAPA Library Quantification Kit (KAPA Biosystems Inc., Wilmington, MA, USA) before amplification with Illumina's cBot. Libraries were sequenced 1 × 50 + 8 bp on Illumina's HiSeq2500.

The bioinformatic analyses to identify miRNA sequences were carried out as follows: the adapter from raw data was removed using a skewer (31), and reads with a size ranging from 15 to 30 bases were aligned to a plant-based reference genome (*Prunus persica*, NCBIv2, and annotation NCBIv2.52 restricted to miRNAs) (hhttps://plants.ensembl.org/Prunus_persica/Info/Index; https://mirbase.org/results/?query=prunus+persica), with miRNA annotation from miRBase (version 22) using ShortStack (32), allowing a maximum of two mismatches in the seed area. Mapped tags were counted using Htseq-count (33), considering the strand information. Raw reads were normalized with DESeq2. Sequencing data were deposited in NCBI's Gene Expression Omnibus (34): GEO series accession number GSE234786 (https://www.ncbi.nlm.nih.gov/geo/query/acc.cgi?acc=GSE234786).

### 2.3 Bioinformatic target gene prediction analysis

The RNA target analysis servers psRNATarget scoring schemas V1 and V2 (https://www.zhaolab.org/psRNATarget/) (35) and TAPIR (http://bioinformatics.psb.ugent.be/webtools/tapir/) (36) were used to predict human putative target genes of all the plant-derived miRNAs identified by NGS.

The cDNA library chosen for both psRNATarget and TAPIR servers was the “*Homo sapiens* (human), transcript, Human genomic sequencing project,” available at psRNATarget, and the bioinformatic predictions were performed with the default parameters. Applying psRNATarget scoring schemas V1 and V2, the following parameters for each output were reported: target accession number of the putative target gene, expectation (penalty for the mismatches), mRNA target aligned fragment, and the inhibitory effect (cleavage or translation inhibition) (35). The scoring Schema V1 also showed the unpaired energy (UPE), which is the energy required to open a mRNA secondary structure (35). The results showed applying the TAPIR program includes the score (penalty for mismatches, gaps, and G-U pairs), the minimum free energy (MFE) ratio, which is a ratio between the free energy of the miRNA–mRNA duplex vs. the free energy of this duplex with perfect matches, and the mRNA target aligned fragment (36). The GeneCodis4 program (https://genecodis.genyo.es/) was used to perform the Gene Ontology (GO) biological process analysis of candidate putative target genes.

### 2.4 miRNA miR482f and miR482c-5p expression analysis

Expression of miR482f and miR482c-5p was analyzed using qPCR in total RNA isolated from 0.1 g of fruits (apple, orange, and pear), 0.1 g of vegetables and greens (green pepper, spinach, raw and cooked green beans, lettuce, and tomato), 0.05–0.1 g of fats and oils (0.05 g of walnut and 0.1 g of olive), 0.1 g of cooked legumes (chickpeas and lentils), 0.2 g of cooked cereals (rice), biological (serum and feces) human samples (described in MM Section 2.8), and cell culture samples (described in MM Section 2.5). Expression of the spike-in UniSp4 was used as a positive control in plant-derived food, and human samples to determine that short RNA fragments/miRNAs were unaffected by RNA isolation and expression analysis. miR-141-3p and miR-103a-3p were used as human (internal) controls to determine the quality of serum and feces samples and the reliability of the results. Previous evidence has reported consistent presence and stability of miR-141-3p and miR-103a-3p in the feces and serum of mammals, including humans (37–40).

Total RNA (4 μl) was reverse transcribed in 10 μl of final volume reaction with miRCURY LNA RT Kit (Qiagen). Reactions were performed in a MyCycler Thermal Cycler (Bio-Rad, Hercules, CA, USA) at 42°C for 60 min and 95°C for 5 min. cDNA from plant-derived food samples was centrifuged for 1 min at maximum speed, and the supernatant was collected for qPCR.

qPCR was performed with cDNA diluted 1/10 using the miRCURY LNA SYBR Green PCR Kit (Qiagen) and miRCURY LNA miRNA PCR Assays (Qiagen). The sequences of the PCR assays are described in Table 1. qPCR was performed in a CFX384 Touch Real-Time PCR Detection System (Bio-Rad) with the following cycling conditions: 95°C for 2 min, 40 cycles at 95°C for 10 s, and 56°C for 1 min. Non-template control samples were added to each qPCR reaction. qPCR duplicates were made for each sample.

**Table 1:** Assays used for qPCR miRNA expression analyses.

**miRNA name**	**miRBase accession number**	**Assay reference (GenGlobe ID)**	**Assay sequence (5^′^-3^′^)**
hsa-miR-141-3p	MIMAT0000432	YP00204504	5′-UAACACUGUCUGGUAAAGAUGG-3′
hsa-miR-103a-3p	MIMAT0000101	YP00204063	5′-AGCAGCAUUGUACAGGGCUAUGA-3′
ppe-miR482f	MIMAT0031512	YP02105458	5′-UCUUUCCUACUCCACCCAUUCC-3′
ppe-miR482c-5p	MIMAT0027283	YP02114485	5′GGAAUGGGCUGUUUGGGAUG-3′
UniSp4	N/A	YP00203953	N/A

MiRNA names, accession number, and sequences of miRCURY LNA miRNA PCR Assays (Qiagen). N/A, non-applicable.

### 2.5 Cell culture and edible plant-derived miRNA mimic transfection

Human monocytic leukemia cell line THP-1 was purchased from the American Type Culture Collection (ATCC^®^ TIB-202™; Manassas, VA, USA). THP-1 cells were maintained in RPMI-1640 medium (Gibco, Thermo Fisher Scientific Inc.) supplemented with 10% fetal bovine serum (FBS; Gibco) and 1% penicillin–streptomycin solution (P/S; Gibco) and incubated at 37°C and 5% CO_2_.

For the experiments, THP-1 cells were seeded in 12-well plates (200,000 cells/well) for gene and miRNA expression assays and 48-well plates (50,000 cells/well) for cytotoxicity assays. The cells were seeded in RPMI-140 medium supplemented with 10% FBS, 1% P/S, and phorbol 12-myristate 13-acetate (PMA; Sigma-Aldrich, San Luis, MO, USA) at a final concentration of 50 ng/ml to induce the differentiation of THP-1 monocytes into macrophages. The cells were incubated for 48 h.

After differentiation, the cells were forward transfected with Lipofectamine RNAiMAX Reagent (Thermo Fisher Scientific Inc., Waltham, MA, USA) and 60 nM of mirVana™ miRNA mimics (Thermo Fisher Scientific Inc.): miR482f (5′-UCUUUCCUACUCCACCCAUUCC-3′), miR482c-5p (5′-GGAAUGGGCUGUUUGGGAUG-3′), and a scramble control (mirVana™ miRNA mimic, negative control #1). Cell medium was changed to RPMI-140 supplemented with 10% FBS, without antibiotics (1 ml/well of 12-well plates, 250 μl/well of 48-well plates). MiRNA mimics and Lipofectamine were diluted in Opti-MEM I Reduced Serum Medium (Gibco), mixed in a 1:1 ratio, and incubated for 5 min at room temperature. miRNA mimic–Lipofectamine complexes were added to each well (100 μl/well of 12-well plates and 25 μl/well of 48-well plates). The volumes of Lipofectamine used were: 2.5 μl per well of 12-well plates (on a volume of 1,100 μl) and 0.625 μl per well of 48-well plates (on a volume of 275 μl). The cells were incubated for 6 h to evaluate the transfection efficiency (detection of transfected miRNAs in cell cultures) or 48 h for gene expression and cytotoxicity assays.

### 2.6 Gene expression analysis

Cells were frozen on dry ice and stored at −80°C. For miRNA expression analysis experiments, before freezing, the cells were washed with PBS. Total RNA was isolated with TRIzol Reagent (Thermo Fisher Scientific Inc.), according to the manufacturer's instructions. RNA quantity and purity (260/280 ratio) were determined in a NanoDrop ND-1000 spectrophotometer (Thermo Fisher Scientific Inc.). miRNA miR482f and miR482c-5p expression analyses were performed as described in MM Section 2.4.

For mRNA expression analysis, RNA (1 μg) was treated with DNA-free™ DNA Removal Kit (Invitrogen. Thermo Fisher Scientific Inc.) following the manufacturer's protocol. DNA-free RNA was reverse transcribed into cDNA with dNTP Mix (Bioline, Luckenwalde, Germany), Random Primers (Invitrogen. Thermo Fisher Scientific Inc.), Recombinant RNAsin Ribonuclease Inhibitor (Promega, Madison, WI, USA), and M-MLV Reverse Transcriptase (Invitrogen). All the reactions were performed in a GeneAmp PCR System 2700 (Applied Biosystems. Thermo Fisher Scientific Inc.). qPCR was performed with cDNA diluted 1/1.5 using iTaq™ Universal Probes Supermix (Bio-Rad) and specific TaqMan^®^ Gene Expression Assays (Thermo Fisher Scientific Inc.) and Predesigned qPCR Assays from Integrated DNA Technologies (Coralville, IA, USA) described in Table 2. Amplification reactions were performed in a CFX384 Touch Real-Time PCR Detection System (Bio-Rad) as follows: 50°C for 2 min, 95°C for 10 min, 40 cycles at 95°C for 15 s, and 60°C for 1 min. Non-template control samples were added to each qPCR reaction. Gene expression levels (Cq) were normalized (ΔCq) with the housekeeping gene TATA box binding protein (*TBP*). The 2^−Δ*ΔCq*^ method (41) was used to determine the relative gene expression of genes, through comparisons between the plant-derived miRNA mimic treatments and the scramble control. At least, two independent experiments were conducted, and qPCR was run in triplicates for each sample.

**Table 2:** Assays used for qPCR gene expression analyses.

**Gene name**	**Assay ID**	**Accession number (RefSeq)**
*NFAM1*	^1^Hs.PT.58.45296389	NM_145912(1)
*CLEC7A*	^1^Hs.PT.58.3686547.g	NM_022570(6)
*TLR6*	^1^Hs.PT.58.26737272.g	NM_006068(1)
*BCL2L12*	^1^Hs.PT.58.4708679	NM_001040668(2)
*IL10*	^2^Hs00961622_m1	NM_000572.2
*TNF*	^2^Hs00174128_m1	NM_000594.3
*IL1B*	^2^Hs01555410_m1	NM_000576.2 XM_017003988.1
*TBP*	^2^Hs00427620_m1	NM_001172085.1 NM_003194.4

Gene names, assay IDs, and accession numbers of Integrated DNA technologies Predesigned qPCR Assays^1^ and TaqMan^®^ Gene Expression Assays^2^.

### 2.7 Cytotoxicity assays

Cell viability and proliferation after miRNA treatments were determined with the (3-4,5-dimethylthiazol-2-yl)-5-(3-carboxymethoxyphenyl)-2-(4-sulfophenyl)-2H-tetrazolium, inner salt (MTS) assay (CellTiter 96^®^ Aqueous One Solution Cell Proliferation Assay. Promega) in THP-1 macrophages. Cells were incubated with MTS reagent (20 μl of reagent per 100 μl of culture medium) for 2.5 h at 37°C. The absorbance was measured at 490 nm in an absorbance microplate reader Agilent BioTek 800 TS (Agilent Technologies). The cell viability was expressed as the % relative viability; calculated as the mean percentage of each experimental group relative to the mean of the scramble control. Four independent experiments were conducted.

### 2.8 Detection of edible plant-derived miR482f and miR482c-5p in biological human samples

Plant-derived miRNA analyses from serum and fecal samples were performed as a proof of concept from anonymous volunteers (>18 yr), who qualitatively consumed plant-based products. The samples were donated by nine volunteers (five men and four women) anonymously, and no personal data were collected from these subjects. Specifically, volunteers usually consumed these plant-based food groups: legumes, fruits, vegetables, cereals, and fats and oils, in the variety and proportion they preferred.

Blood samples were collected through a venous puncture in BD Vacutainer^®^ SST™ II Advance tubes (BD Vacutainer Systems, Plymouth, United Kingdom). Samples were kept at room temperature for 30 min and centrifuged at 2,000 *g* for 15 min at 4°C. Serum was collected and centrifuged at 16,000 *g* for 10 min at 4°C. Total RNA was extracted from 200 μl of serum samples using miRNeasy Serum/Plasma Advanced Kit (Qiagen). Fecal samples were collected in stool nucleic acid collection and preservation tubes (Norgen Biotek Corp., ON, Canada) according to the manufacturer's instructions. Total RNA was isolated using the RNeasy PowerMicrobiome Kit (Qiagen) from 250 μl of feces following the manufacturer's protocol. Overall, 1 μl of spike-in controls (Qiagen) was added to the serum and fecal samples before RNA extraction. MiRNA miR482f and miR482c-5p expression analyses were performed as described in MM Section 2.4.

### 2.9 Statistical analysis

The statistical analyses were performed using GraphPad Prism 6.0 for Windows (GraphPad Software Inc., La Jolla, CA, USA). For *in vitro* studies, comparisons between the miRNA negative control and the experimental groups were performed using Student's *t*-test. Student's *t*-test was selected because the aim was to determine differences between plant-derived miR482f and miR482c-5p with the negative control, as independent studies, and not to establish a comparison between the three groups. Statistical significance was considered at *p-*value <0.05.

## 3 Results

### 3.1 Plant-derived miRNAs are present in edible plant-based products, and miR482f and miR482c-5p could potentially inhibit the expression of pro-inflammatory human genes

We performed NGS analysis to identify miRNAs in edible plant-based foods, including fruits (apple, orange, and pear), vegetables (spinach and tomato), and fats and oils (walnut and olive). After the alignment of the reads with the peach genome, as a reference genome, 176 miRNAs were identified in the overall pool of plant-based food samples analyzed (Supplementary Table S1).

The bioinformatic analysis with psRNATarget and TAPIR predicted potential human target genes of the 176 miRNAs detected in the plant-based food samples (data not shown). In the present study, we only focused on miR482f (Ensembl gene: ENSRNA049996209) and miR482c (Ensembl gene ENSRNA049996546) because human gene prediction analyses revealed their potential role in targeting inflammation-related human genes.

The alignment of the miR482f mature sequence with the human transcriptome revealed that 6 (psRNATarget scoring schema V1), 24 (psRNATarget scoring schema V2), and 5 (TAPIR) transcripts could be putative targets (Supplementary Table S2). Altogether, 26 different transcripts (corresponding to 22 different genes) were predicted as putative targets of miR482f. Only three transcripts appeared in the three algorithms. These were as follows: the transcript NM_145912, which codifies for NFAT activating protein with ITAM motif 1 (NFAM1), and the transcripts NM_197949 and NM_022570, which codify for two isoforms of the C-type lectin domain containing 7A (CLEC7A) protein (Supplementary Table S2, Supplementary Figure S1).

The alignment of the plant-derived miR482c-5p mature sequence and the human transcriptome reported that 2 (psRNATarget scoring schema V1), 24 (psRNATarget scoring schema V2), and 5 (TAPIR) transcripts could be putative target genes (Supplementary Table S3). A total of 27 different transcripts (22 different genes) were identified as putative targets of plant-derived miR482c-5p. The only common targets of the three prediction programs were the transcript NM_001160332 of the neurofascin (NFASC) protein and the transcript NM_006068, which encodes for the Toll-like receptor 6 (TLR6) (Supplementary Table S3, Supplementary Figure S2).

According to the bibliography, the combination of psRNATarget and TAPIR programs gives highly accurate predictions (42). Therefore, we selected for subsequent analyses the common target genes to psRNATarget (schemas V1 and V1) and TAPIR: *NFAM1* and *CLEC7A* as putative target genes of miR482f, and *NFASC* and *TLR6* as putative target genes of miR482c-5p.

To explore the biological functions shared between the predicted target genes, we performed Gene Ontology (GO) biological process analysis with GeneCodis4 (Supplementary Figure S3). For the miR482f potential targets, the results revealed that both *NFAM1* and *CLEC7A* participate in the inflammatory response and in the positive regulation of DNA-binding transcription activity (Supplementary Figure S3A). GeneCodis4 associated *TLR6* with a wide variety of functions related to the immune system, such as macrophage activation, and pro-inflammatory cytokines production (Supplementary Figure S3B). We did not delve into the study of the potential role of miR482c-5p in shaping the *NFASC* gene expression since the aim of the present study was to evaluate the impact of plant-derived miRNAs on inflammation. *NFASC* main functions are related to the nervous system (43). According to Protein Atlas (https://www.proteinatlas.org/ENSG00000163531-NFASC/subcellular), its expression is negligible in THP-1 monocytes, which was the *in vitro* model selected in this study to achieve the abovementioned objective.

Regarding miR482f, we identified 220.38 normalized reads in the walnut sample, which were also detected in the tomato, orange, olive, and pear samples (Table 3). miR482c, which comprises−5p and−3p sequences, was mostly detected in the walnut and pear samples (655.13 and 266.26 normalized reads, respectively) as well as in the apple, orange, spinach, and olive samples (Table 3), although in lower copy read numbers.

**Table 3:** Detection of miR482f and miR482c in plant-based foods by next-generation sequencing.

**Gene ID**	**miRNA name**	**Normalized reads**						
		**Walnut**	**Tomato**	**Orange**	**Pear**	**Apple**	**Olive**	**Spinach**
gene:ENSRNA049996209	miR482f	220.38	78.09	45.35	0.10	U	0.41	U
gene:ENSRNA049996546	miR482c	655.13	U	0.60	266.26	67.70	0.28	0.27

Small RNA-seq was performed with total RNA isolated from 0.05 to 0.1 g of fats and oils and 0.1 g of fruits and vegetables. Reads were mapped to the *Prunus persica* genome (annotation NCBIv2.52). Results correspond to the normalized reads of a single sample per plant-based product. U, undetectable.

**Figure 1:**
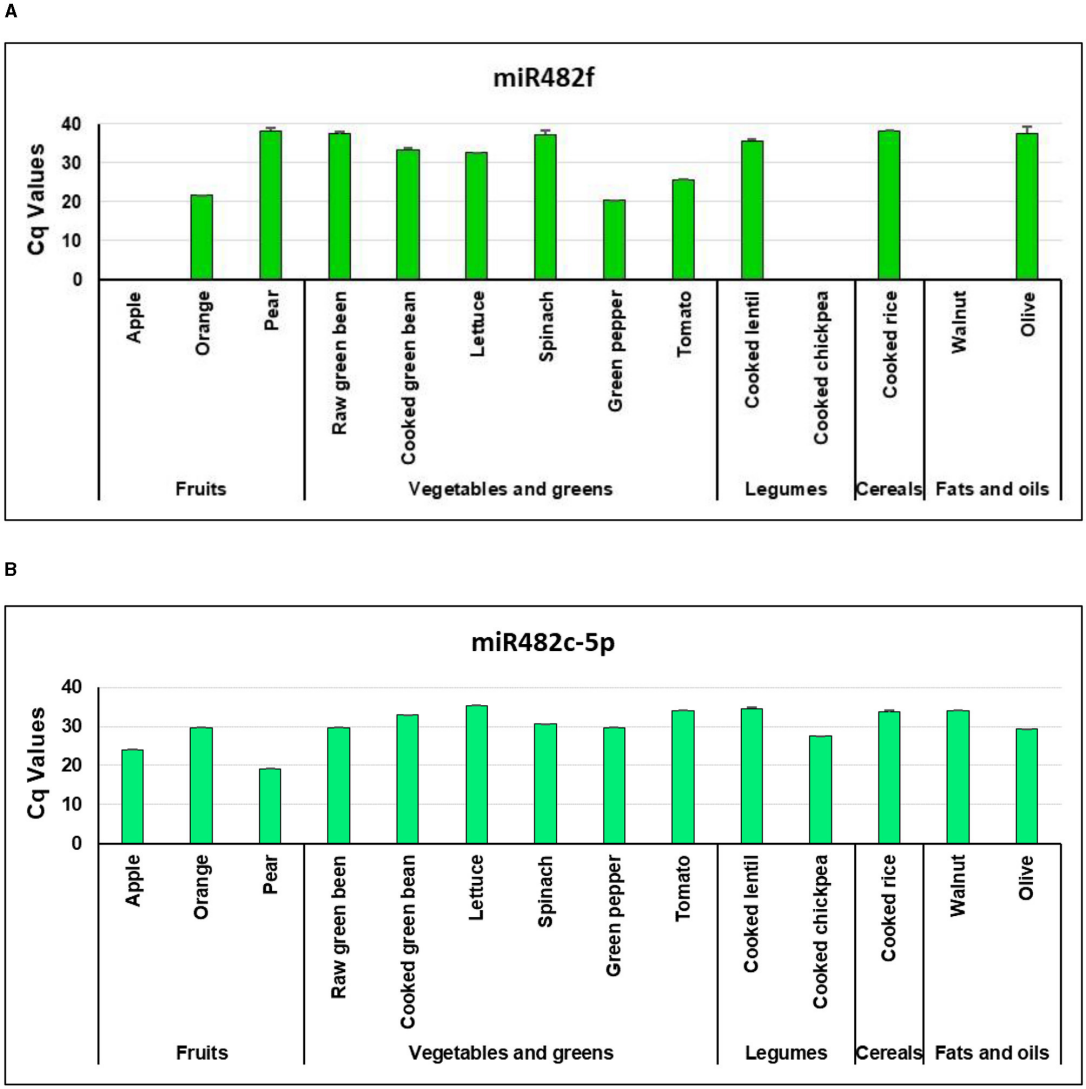
Detection of miR482f and miR482c-5p in plant-based foods by qPCR. Cq values of **(A)** miR482f (5′-UCUUUCCUACUCCACCCAUUCC-3′) and **(B)** miR482c-5p (5′-GGAAUGGGCUGUUUGGGAUG-3′) with RNA isolated from 0.1 g of fruits (apple, orange, and pear), 0.1 g of vegetables and greens (green pepper, spinach, raw and cooked green beans, lettuce, and tomato), 0.05–0.1 g of fats and oils (0.05 g of walnut and 0.1 g of olive), 0.1 g of cooked legumes (chickpea and lentil), and 0.2 g of cooked cereals (rice). Results are the mean ± standard error of the mean (SEM) (one sample per type of food product and duplicates for qPCR).

We next sought to qualitatively complement the small RNA-seq results using qPCR analysis in different food matrices, including a greater range of edible plant-derived foods: fruits (apples, oranges, and pears), vegetables and greens (green pepper, spinach, raw and cooked green beans, lettuce, and tomato), fats and oils (walnuts and olives), cooked legumes (chickpeas and lentils), and cooked cereals (rice) (Figure 1). miR482f was detected in fruits (orange and pear), vegetables and greens (raw and cooked green beans, lettuce, spinach, green pepper, and tomato), legumes (cooked lentils), cereals (cooked rice), and fats and oils (olive) (Figure 1A). Regarding miR482c, we validated that the sequence corresponding to the transcript miR482c-5p. miR482c-5p was present in all the selected edible plant-derived foods (Figure 1B). The objective of this analysis was to determine the presence or absence of miRNAs, rather than comparing their abundance between samples. Therefore, data were not normalized to any small RNA sequence (housekeeping) as usually performed for qPCR methods, providing that (1) our aim required only a qualitative measurement and (2) no standardized normalization method has been established for plant-derived miRNA quantification. Positive detection of the spike-in UniSp4 was achieved in all food-based samples, which confirmed the accurate isolation and detection of miRNAs (data not shown).

Overall, miR482f and miR482c-5p were present in the different raw and cooked plant-derived foods that were analyzed. The miRNA target gene prediction algorithms and the Genecodis4 GO biological processes analyses, suggest that miR482f and miR482c-5p could potentially inhibit the expression of genes involved in inflammation. Therefore, we delved into the study of the potential role of miR482f and miR482c-5p modulating the expression of their putative target genes (*NFAM1* and *CLEC7A*, and *TLR6*, respectively), and their potential impact on the expression profile of anti- and pro-inflammatory cytokines, in an *in vitro* model of human macrophages.

### 3.2 Plant-derived miR482f and miR482c-5p mimics are present in human macrophage-like THP-1 cells after transfection

Human monocytes THP-1 cells differentiated to macrophages were transfected with miRNA mimics miR482f and miR482c-5p, and a scramble control at a concentration of 60 nM. To determine the effectiveness of transfections, we performed qPCR analysis to detect the miRNAs after 6 h of transfection (Supplementary Figure S4). miR482f and miR482c-5p were present in THP-1 cells transfected with each of these mimics (Cq values between 12 and 13). The plant-derived miRNA sequences were not detected in negative control-transfected cells, suggesting that miR482f and miR482c-5p have no human homologous miRNAs in the THP-1 cells.

### 3.3 Impact of plant-derived miR482f and miR482c-5p mimics on macrophage-like THP-1 viability and/or proliferation

**Figure 2:**
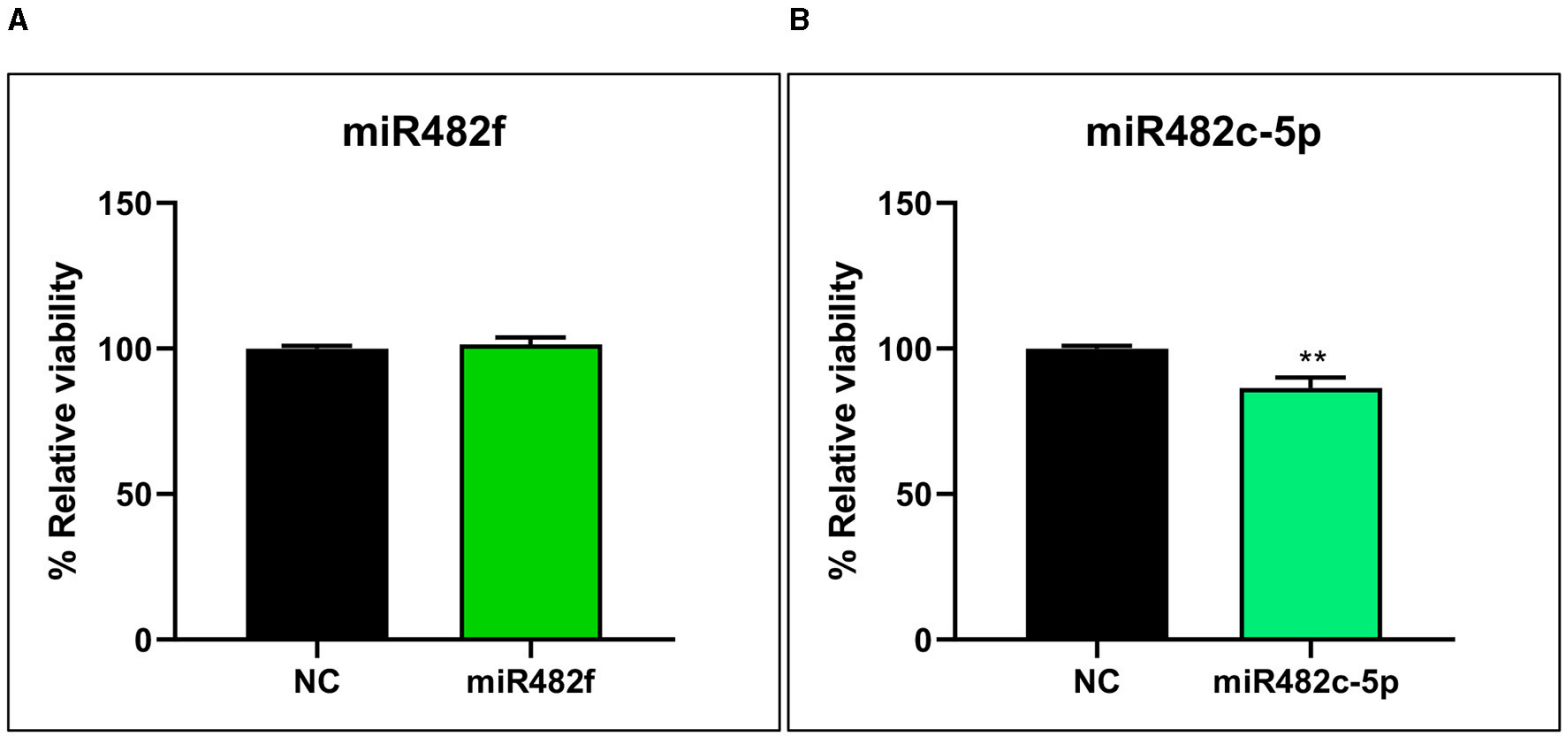
Evaluation of the effect of plant-derived miRNA mimics **(A)** miR482f and **(B)** miR482c-5p on the viability/proliferation of THP-1 macrophage-like cells. THP-1 monocytes differentiated to macrophages for 48 h were transfected with 60 nM of miRVana mimics miR482f (5′-UCUUUCCUACUCCACCCAUUCC-3′), miR482c-5p (5′-GGAAUGGGCUGUUUGGGAUG-3′), and a scramble sequence as a negative control (NC). Cell viability/proliferation was evaluated in THP-1 macrophage-like cells after 48 h of transfections. Results are the mean percentage ± standard error of the mean (SEM) relative to the cells transfected with the scramble sequence (negative control) (*n* = 4 independent experiments; NC: *n* = 13; miR482f: *n* = 13; miR482c-5p: *n* = 13). NC, negative control. Significance refers to the effect of miR482f or miR482c-5p with respect to the negative control. *p*-value: ** *p* < 0.01.

The viability/proliferation of THP-1 macrophage-like cells was evaluated with MTS at 48 h of transfection with 60 nM of plant-derived miR482f, miR482c-5p mimics, and a scramble sequence as a control (Figure 2). miR482f mimic did not induce any change in cell viability or proliferation (Figure 2A), whereas miR482c-5p mimic decreased 13.5 ± 3.6% of the viability/proliferation of THP-1-like macrophages in comparison with the control cells (Figure 2B).

### 3.4 Plant-derived miR482f and miR482c-5p modulate gene expression of predicted target genes and inflammation-related biomarkers in THP-1 cells

We validated the human target gene bioinformatic predictions (*NFAM1* and *CLEC7A* for miR482f; and *TLR6* for mi482c-5p) in the transfected THP-1 macrophage-like cells. Gene expression profile of anti- and pro-inflammatory biomarkers (interleukin 10, *IL10*; tumor necrosis factor, *TNF*; interleukin 1 Beta; and *IL1B*) and the Bcl-2-like protein 12 (*BCL2L12*) was evaluated.

**Figure 3:**
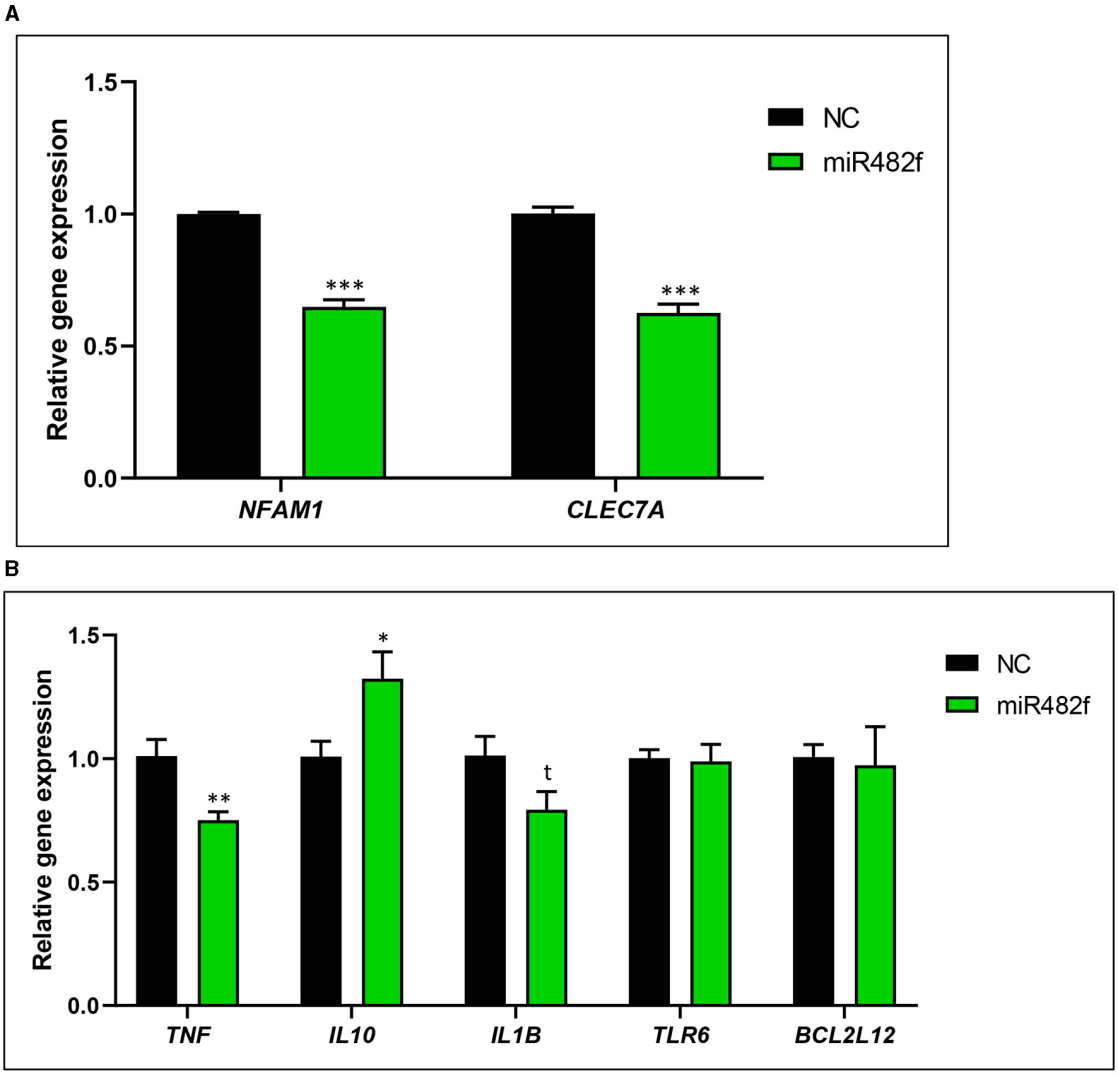
Gene expression analysis of THP-1 macrophage-like cells transfected with plant-derived miR482f mimic. **(A)** mRNA levels of putative targets *NFAM1* and *CLEC7A*. **(B)** Gene expression levels (mRNA) of pro-inflammatory (*TNF, IL1B*, and *TLR6*) and anti-inflammatory (*IL10*) biomarkers and *BCL2L12*. THP-1 macrophage-like cells were transfected with 60 nM of miRVana mimic miR482f (5′-UCUUUCCUACUCCACCCAUUCC-3′) and a scramble sequence as a negative control. Gene expression levels were measured at 48 h of transfection by qPCR. Results are the gene expression levels of each gene normalized to the housekeeping gene *TBP* and calculated by the 2^−Δ*ΔCt*^ method, establishing comparisons with the negative control. NC, negative control. The results are presented as mean percentage ± standard error of the mean (SEM) (*n* = 2 independent experiments, run in triplicate; NC: *n* = 5; miR482f: *n* = 6). *p*-value: * *p* < 0.05, ** *p* < 0.01, *** *p* < 0.001, t = 0.0708.

miR482f mimic downregulated the gene expression of the putative targets *NFAM1* (35.2% ± 3.0; *p* < 0.0001) and *CLEC7A* (37.7% ± 4.2; *p* < 0.0001) (Figure 3A) after 48 h of miRNA transfection. miR482f mimic increased the mRNA levels of the anti-inflammatory cytokine *IL10* (31.5% ± 13.3, *p* < 0.05) and reduced the mRNA levels of the pro-inflammatory cytokine *TNF* (26.0% ± 7.2, *p* < 0.01) (Figure 3B). No significant changes were detected for *IL1B, TRL6*, and *BCL2L12* gene expression (Figure 3B). A trend was observed for *IL1B* mRNA decrease (21.9 % ± 10.7, *p* = 0.0708).

**Figure 4:**
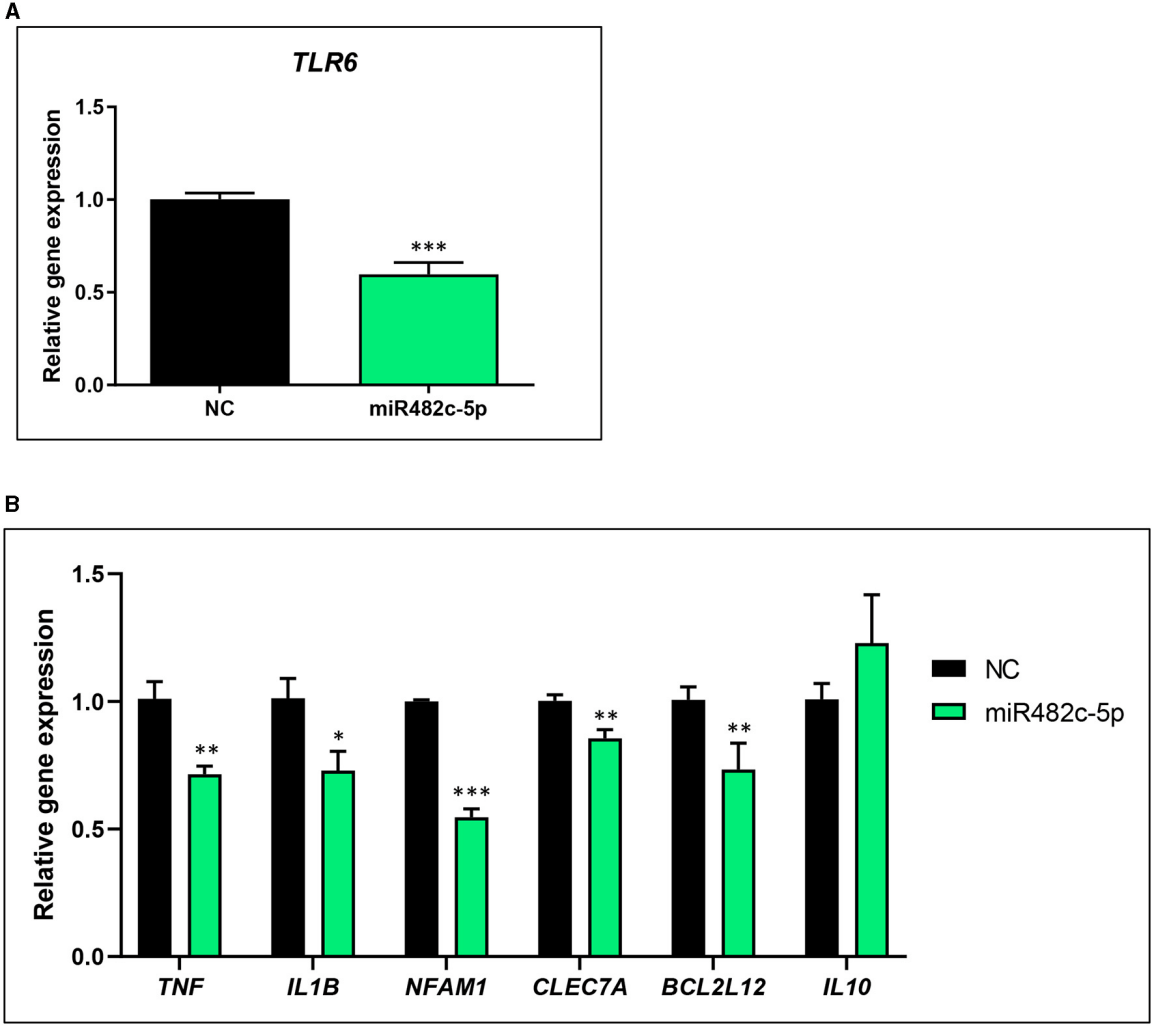
Gene expression analysis of THP-1 macrophage-like cells transfected with plant-derived miR482c-5p mimic. **(A)** mRNA levels of putative target *TLR6*. **(B)** Gene expression levels (mRNA) of pro-inflammatory (*TNF, IL1B, TLR6*) and anti-inflammatory (*IL10*) biomarkers and *BCL2L12*. THP-1 macrophage-like cells were transfected with 60 nM of miRVana mimic miR482c-5p (5′-GGAAUGGGCUGUUUGGGAUG-3′) and a scramble sequence as a negative control. Gene expression levels were measured at 48 h of transfection by qPCR. Results are the gene expression levels of each gene normalized to the housekeeping gene *TBP* and calculated by the 2^−Δ*ΔCt*^ method, establishing comparisons with the negative control. NC, negative control. The results are presented as mean percentage ± standard error of the mean (SEM) (*n* = 2 independent experiments, run in triplicate; NC: *n* = 5; miR482f: *NFAM1, TNF, n* = 5; *CLEC7A, BCL2L12, IL10, IL1B, n* = 6). *p*-value: * *p* < 0.05, ** *p* < 0.01, *** *p* < 0.001. Plant-derived miRNAs miR482f and miR482c-5p are detected in human feces but not in serum.

The transfection of miR482c-5p mimic reduced the mRNA levels of the putative target gene *TLR6* (40.5% ± 7.6 %, *p* < 0.001) at 48 h of transfection (Figure 4A). miR482c-5p downregulated gene expression of the pro-inflammatory genes *TNF* (29.6% ± 7.5, *p* < 0.01), *NFAM1* (45.4% ± 3.4, *p* < 0.0001), *CLEC7A* (14.7% ± 4.4 *p* < 0.01), *IL1B* (28.4% ± 10.93 *p* < 0.05), and *BCL2L12* (27.39% ± 8.2, *p* < 0.01), but it did not induce significant changes in the expression of the anti-inflammatory cytokine *IL10* (Figure 4B).

### 3.5 Plant-derived miRNAs miR482f and miR482c-5p are detected in human feces but not in serum

To determine whether miR482f and miR482c-5p derived from plant-based foods could reach the gastrointestinal tract, resist degradation during digestion, and potentially be absorbed, we collected feces and serum samples from healthy volunteers who usually consume edible plant-based products. The positive expression of the spike-in UniSp4 in both serum and feces samples confirmed the accuracy of the experimental procedure (RNA isolation and expression analysis) (data not shown).

**Figure 5:**
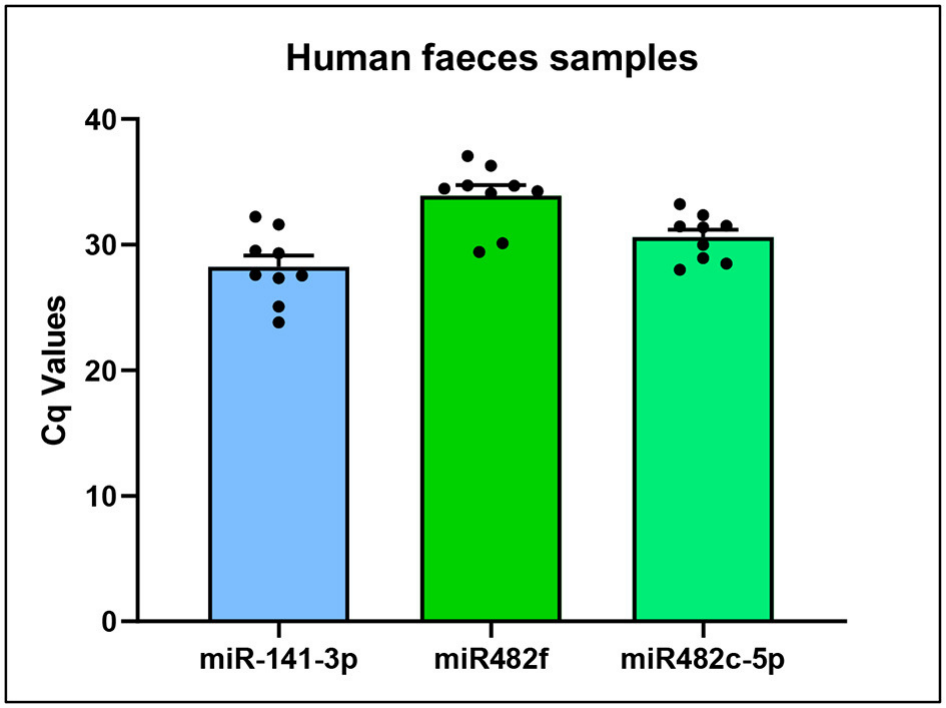
Detection of plant-derived miR482f and miR482c-5p in human feces of healthy volunteers using qPCR. Cq values of plant-derived miR482f (5′-UCUUUCCUACUCCACCCAUUCC-3′), miR482c-5p (5′-GGAAUGGGCUGUUUGGGAUG-3′), and human miR-141-3p (5′-UAACACUGUCUGGUAAAGAUGG-3′) as an endogenous control, in nine human feces samples (qPCR duplicates for each sample) from healthy volunteers. Results are the mean ± standard error of the mean (SEM) (*n* = 9).

We measured expression levels of plant-derived miR482f and miR482c-5p using qPCR in nine fecal samples (Figure 5). Both miR482f and miR482c-5p were detected in all the samples, suggesting that plant-derived miRNAs are present in the gastrointestinal tract and resist digestion. The expression of the human hsa-miR-141-3p was used as an endogenous control to determine the quality of the fecal samples.

We analyzed the levels of plant-derived miR482f and miR482c-5p in serum samples from the same volunteers in which the two plant-derived miRNAs were present in feces. Neither miR482f nor miR482c-5p were detected in serum samples in any of the volunteers (data not shown). To further confirm the reliability of the results, we used the human miR-103a-3p as an endogenous control. hsa-miR-103a-3p was detected in all the serum samples analyzed (Cq values between 24 and 28), suggesting that the lack of detection of plant-derived miR482f and miR482c-5p could be due to their very low or inexistent quantity to be detected by qPCR and not because of the serum sample quality.

## 4 Discussion

The present study aimed to identify miRNAs from edible plant-derived foods that could modulate the expression of human genes associated with inflammation. The rationale of this hypothesis is mainly based on the evidence supporting (1) the role of plant-based diets in alleviating inflammation, and (2) the recent discovery of miRNAs as bioactive molecules of plants and cross-kingdom gene expression regulators (19, 44).

We selected five groups of plant-derived foods (fruits, vegetables and greens, legumes, cereals, and fats and oils) based on previous evidence that unveiled their anti-inflammatory properties (8–11). Although we identified a wide variety of miRNAs in edible plants, miR482f and miR482c-5p were selected for their potential role in targeting inflammatory human genes. We found that these miRNAs are present in the five groups of foods. We used the *Prunus Persica* genome as a reference to annotate plant-derived miRNA sequences of edible plants detected by NGS. As regards our results, both NGS and qPCR, there is a high degree of conservation of these miRNAs in the plant kingdom or at least among the plant-origin food products analyzed in this study. The results revealed that original plant-derived miR482f and miR482c withstand standard heat-treatment (cooking) processes. In agreement with our findings, other authors have also reported that harsh conditions such as storage, processing, and cooking do not compromise plant-derived miRNA survival (45). For some plant-based food samples, we detected miR482f or miR482c by NGS and not by qPCR and vice versa. For instance, we detected miR482f in walnuts only by NGS and in spinach only by qPCR. The miR482c were detected in tomato only by qPCR. The results obtained with both techniques are complementary, given that the nature of the sample might determine the suitability for either NGS and/or qPCR. However, the NGS hits that could not be validated by qPCR should be taken with caution. Reliable conclusions can only be drawn about the presence or absence of miR482f and miR482c in each plant-based product (verified by two independent methods). The relative abundance between different types of plant-based products cannot be compared since there is no reliable and universal endogenous plant-derived miRNA normalization method established yet. Our *in silico* analysis predicted that our miRNA candidates (miR482f and miR482c-5p) would eventually bind specific mRNA targets associated with inflammation. Providing that the mechanism of action of miRNA usually links to mRNA inhibition (target repression), plant-derived miR482f and miR482c-5p could eventually downregulate inflammation-related genes. To eventually validate this hypothesis, we used differentiated human THP-1 macrophages, an *in vitro* cell model widely used in the study of the antioxidant and anti-inflammatory bioactive properties of chemical compounds and derived food products (46–48).

Using differentiated human THP-1 macrophages, we have demonstrated for the first time that plant-derived miR482f and miR482c-5p can achieve efficient inhibition of their predicted targets (*CLEC7A* and *NFAM1* and *TLR6*, respectively) and induce significant changes in other (functional) inflammation-related genes. As regards our results, plant-derived miR482f and miR482c-5p stand out as bioactive molecules from dietary sources with the potential to manage inflammation-associated genes. However, specific differences (in direct targets and in functional genes) between both miRNAs exist and could suggest different but complementary mechanisms of action for each plant-derived miRNA. miR482f and miR482c-5p direct targets had previously been linked to immune system function (49–52) and production of pro-inflammatory cytokines (such as TNF-α, IL1-β, and IL-6) (53–56), suggesting that their inhibition could have a therapeutic approach. The finding that miR482c-5p also reciprocally downregulated *CLEC7A* and *NFAM1* gene expression as its counterpart (miR482f) is of relevance since a positive correlation between *TLR6* and *CLEC7A* expression has also been reported (57). On the other hand, while miR482f reciprocally upregulated anti-inflammatory (*IL10*) and downregulated pro-inflammatory factors (*TNF*), miR482c-5p only downregulated pro-inflammatory genes (*TNF, IL1B*). Whether these changes at the mRNA expression level translate to functional (i.e., protein secretion) modifications remains to be established in future studies.

Interestingly, it has been documented that TLRs could modulate the expression of anti-apoptotic Bcl-2 family proteins, and TLR6 could protect cells from apoptosis (58–61). This evidence could explain the slight decrease in the viability/proliferation that we detected upon the inhibition of *TLR6* by miR482c-5p. In concordance, the downregulation of the anti-apoptotic gene *BCL2L12* (62, 63), in miR482c-5p treated cells suggests that this miRNA could be interfering with the viability of macrophages. We hypothesize that, since in miR482c-5p-treated cells the *TLR6* levels decrease, these cells are less protected against pro-apoptotic stimuli than other (scramble control and miR482f-treated) cells in which the expression of *TLR6* was not affected. We speculate that *TLR6* inhibition might not decrease cell viability in physiological conditions absent of potentially harmful events (i.e., transfection procedure). In any case, we consider that the decrease in macrophage viability (10%) is very low to be considered biologically relevant.

The role of plant-derived miRNAs as cross-kingdom immune-modulatory agents has recently been documented. This could be mediated through plant-derived miRNA interaction with viral, bacteria, and mammalian cell genes (64–68). In the present study, we reported a direct interaction between plant-derived miRNAs with immunomodulatory properties and mammalian genes, which has been also documented by other studies (67, 68). Indeed, we found that plant-derived miRNAs could interact with immune system cells, in particular with human macrophages, and regulate gene expression. Although the mechanism underlying the interactions between plant-derived miRNAs and immune system cells described in our study relies mainly on gene expression regulation, we do not exclude the involvement of other additional/complementary mechanisms (i.e., involving indirect target gene interactions) (68).

A crucial but controversial aspect of the plant-derived miRNA cross-kingdom gene expression regulation hypothesis is the bioavailability in the host. Interestingly, edible plant-derived miRNAs could be present in extracellular vesicles (EVs) (69, 70), which could be a key determinant of absorption (16, 71, 72). Nevertheless, bioactive activities upon the absorption by mammalian cells have been reported *in vivo* after the administration of plant-derived miRNAs in a free form (68, 73). This evidence suggests that either in a free form or encapsulated in EVs, plant-derived miRNAs could have a cross-kingdom functional impact. In the present study, we verified that plant-derived miR482f and miR482c-5p were detected in human feces but not in the serum of healthy individuals who usually consumed plant-based products. These results demonstrate that plant-derived miRNAs (miR482f and miR482c-5p) resist physical (cooking and digestive motility) and chemical treatments (digestive enzymes) but is rather unlikely that they could reach physiological levels in the bloodstream. We do not rule out completely the possibility that miR482f and miR482c-5p could be absorbed since it is quite reasonable that their bioavailability might be lower (or beyond that) the detection level of standard methods and conditions used for addressing small RNA expression. It could be worthy to analyze their presence in serum in other experimental conditions since the bioavailability of miRNAs might depend on dietary intake levels (74), gut permeability variability (16), and the sensitivity of the techniques used for its detection. Interestingly, it has been suggested that technical limitations, artifacts, contaminations, inter-individual variability in gut permeability, and dietary abundance and stability of plant-derived miRNAs could explain the disparity of the results reported between studies (75, 76). Importantly, plant-derived miRNAs could potentially modulate inflammation at the local (gut) level through the modulation of tissue-resident immune cells (macrophages, lymphocytes) and/or gut microbiota. Although some previous evidence supports this hypothesis (66), intimate mechanisms of this cross-kingdom communication remain to be established; especially, the direct interaction of plant-derived miRNAs with gut immune cells should be confirmed in future *in vivo* and/or clinical studies. The abundance of plant-derived miR482f and miR482c-5p in fecal samples suggests that they reach the gastrointestinal tract (and move forward throughout the intestinal lumen). As regards our findings, plant-derived miRNAs are physically present and stable in the gastrointestinal tract. Whether their presence in host organisms fully translates into physiological changes (at cellular/tissue level) remains to be established in future experiments since it would require additional resources (methodologies, ethical) or invasive procedures (biopsies) and this was out of our scope. Although *in vivo* approaches would eventually contribute to clarify this, important inter-species differences arise. For instance, *in silico* plant-derived miR482f and miR482c-5p predictions are biologically different in human vs. mouse (according to their respective mRNA sequence) and thus prevent the implementation of animal models in the context of our study.

This article is not free of limitations. Additional research would be necessary to explore the role of plant-derived miR482f and miR482c-5p on inflammation beyond the regulation of gene expression levels. To determine if plant-derived miR482f and miR482c-5p effects at the gene level could translate to physiological events such as cytokine protein expression changes, we evaluated IL-10 secretion levels in conditioned media, as a first preliminary (proof of concept) approach. We found that these miRNAs modulated IL-10 secretion levels (data not shown), concordantly to reported gene expression changes. However, depth investigations will be required to evaluate the overall impact of plant-derived miR482f and miR482c-5p on the protein secretion levels of the whole cytokines panel presented in this study (a secretome analysis). Thus, whether the modulation of inflammatory-associated genes promoted by plant-derived miR482f and miR482c-5p fully translate to physiologically relevant events remains to be established in future studies. This could determine whether these miRNAs might potentially be important therapeutic agents to shape immune system responses.

## 5 Conclusion

We show here that miR482f and miR482c-5p are present in standard plant-based foods (legumes, vegetables, greens, fruits, cereals, and fats and oils), resist physical–chemical degradation during cooking and digestion, and reach the human gut, although eventually, they are not absorbed. *In vitro*, these plant-derived miRNAs modulate the gene expression profile of inflammation-associated biomarkers in human macrophages. A better understanding of the cross-talk between plant-derived miR482f and miR482c-5p and the immune system could help to determine their therapeutic potential as bioactive molecules with anti-inflammatory properties.

## Data availability statement

The original contributions presented in the study are publicly available. This data can be found here: https://www.ncbi.nlm.nih.gov/geo/query/acc.cgi?acc=GSE234786.

## Ethics statement

The studies involving humans were approved by University of Navarra, Pamplona, Spain. The studies were conducted in accordance with the local legislation and institutional requirements. The participants provided their written informed consent to participate in this study.

## Author contributions

ED-S: Conceptualization, Methodology, Writing – original draft, Writing – review & editing. SL-C: Conceptualization, Funding acquisition, Project administration, Supervision, Writing – review & editing. PA: Methodology, Writing – review & editing. E-ZA: Writing – review & editing. JR-B: Funding acquisition, Project administration, Writing – review & editing. FM: Conceptualization, Funding acquisition, Project administration, Supervision, Writing – review & editing.
