# Supplementary material for: miR482f and miR482c-5p from edible plant-derived foods inhibit the expression of pro-inflammatory genes in human THP-1 macrophages
Source: Front Nutr. 2023 Nov 30;10:1287312. doi: 10.3389/fnut.2023.1287312 (PMC10719859; doi:10.3389/fnut.2023.1287312)
Supplement: Supplementary file 1 [file Data_Sheet_1.docx]

Supplementary Material

**Table S1.** Detection of miRNAs in plant foods by next-generation sequencing.

| Gene ID | miRNA name | Normalized reads | | | | | | |
| --- | --- | --- | --- | --- | --- | --- | --- | --- |
|  |  | **Walnut** | **Tomato** | **Orange** | **Pear** | **Apple** | **Olive** | **Spinach** |
| gene:ENSRNA049995925 | ppe-MIR6258 | U | 0.05 | U | U | U | 0.14 | U |
| gene:ENSRNA049995957 | ppe-MIR8133 | U | 0.02 | 0.10 | U | U | U | U |
| gene:ENSRNA049995965 | ppe-MIR167d | 3320.78 | 0.48 | 12.06 | 2.04 | 2.00 | 10.94 | 0.60 |
| gene:ENSRNA049995970 | ppe-MIR166a | 49.81 | 28.41 | 1454.71 | 1.84 | 0.78 | 2148.03 | 1.58 |
| gene:ENSRNA049995989 | ppe-MIR7122a | U | 0.02 | 5.08 | 55.99 | 0.04 | 1.64 | U |
| gene:ENSRNA049995996 | ppe-MIR169l | U | U | U | U | U | 0.27 | U |
| gene:ENSRNA049996011 | ppe-MIR169b | 1.04 | 0.05 | 0.50 | 0.03 | U | 21.74 | 0.05 |
| gene:ENSRNA049996020 | ppe-MIR169k | 0.26 | U | U | U | U | 0.14 | U |
| gene:ENSRNA049996028 | ppe-MIR169d | U | 0.02 | U | U | U | U | U |
| gene:ENSRNA049996034 | ppe-MIR169a | 1.04 | U | 0.50 | U | U | U | 0.19 |
| gene:ENSRNA049996042 | ppe-MIR403 | 201.08 | 17.68 | 136.05 | 11.17 | 1.97 | 465.10 | 74.61 |
| gene:ENSRNA049996055 | ppe-MIR169j | U | 0.05 | 0.50 | U | U | 1.09 | 0.23 |
| gene:ENSRNA049996063 | ppe-MIR6262 | U | 0.02 | U | U | U | U | U |
| gene:ENSRNA049996069 | ppe-MIR395m | 0.52 | U | 0.20 | U | 0.25 | U | U |
| gene:ENSRNA049996078 | ppe-MIR395h | U | U | 0.10 | U | 0.00 | U | U |
| gene:ENSRNA049996092 | ppe-MIR395g | U | U | U | U | U | 7.25 | U |
| gene:ENSRNA049996100 | ppe-MIR395l | U | U | U | U | U | 8.07 | 0.42 |
| gene:ENSRNA049996107 | ppe-MIR395a | U | U | 0.20 | U | U | U | U |
| gene:ENSRNA049996115 | ppe-MIR395j | 0.26 | U | U | 0.07 | U | U | U |
| gene:ENSRNA049996122 | ppe-MIR395d | U | 0.18 | U | U | U | 0.14 | U |
| gene:ENSRNA049996130 | ppe-MIR395c | U | 0.76 | U | 0.17 | 0.12 | 6.43 | U |
| gene:ENSRNA049996137 | ppe-MIR395i | U | 0.76 | 0.40 | 0.10 | U | U | U |
| gene:ENSRNA049996145 | ppe-MIR395n | U | U | 0.50 | 0.24 | U | 7.11 | U |
| gene:ENSRNA049996152 | ppe-MIR395o | U | 0.07 | U | 0.03 | U | U | U |
| gene:ENSRNA049996180 | ppe-MIR395f | U | 0.12 | U | 1.84 | U | U | U |
| gene:ENSRNA049996187 | ppe-MIR482a | U | U | 1.30 | 0.24 | 0.02 | 41.29 | U |
| gene:ENSRNA049996202 | ppe-MIR482b | U | U | 3.09 | U | U | 0.27 | U |
| gene:ENSRNA049996209 | ppe-MIR482f | 220.38 | 78.09 | 45.35 | 0.10 | U | 0.41 | U |
| gene:ENSRNA049996219 | ppe-MIR394a | 7.04 | 0.05 | 16.55 | 1.23 | 0.02 | 1.09 | 3.53 |
| gene:ENSRNA049996227 | ppe-MIR171f | 250.89 | 0.02 | 16.35 | U | U | 0.55 | 0.28 |
| gene:ENSRNA049996234 | ppe-MIR156e | 43.29 | U | 8.57 | 65.73 | 2.05 | 10.25 | 48.92 |
| gene:ENSRNA049996241 | ppe-MIR156d | 13.04 | 0.02 | 0.60 | 0.24 | 0.00 | 30.76 | 0.14 |
| gene:ENSRNA049996257 | ppe-MIR396a | U | 0.28 | U | 0.17 | 0.11 | 0.41 | U |
| gene:ENSRNA049996261 | ppe-MIR6260 | U | 0.02 | U | U | U | U | U |
| gene:ENSRNA049996271 | ppe-MIR399k | 0.78 | U | U | U | U | 0.96 | U |
| gene:ENSRNA049996279 | ppe-MIR6261 | U | 0.05 | U | U | U | U | U |
| gene:ENSRNA049996288 | ppe-MIR394b | U | 0.07 | 11.96 | 1.91 | 0.01 | 1.09 | 0.05 |
| gene:ENSRNA049996297 | ppe-MIR6264 | U | U | U | U | U | 0.14 | U |
| gene:ENSRNA049996304 | ppe-MIR8125 | U | 0.02 | 0.10 | 0.07 | U | 1.09 | 0.05 |
| gene:ENSRNA049996310 | ppe-MIR6263 | U | U | U | U | U | U | 0.05 |
| gene:ENSRNA049996337 | ppe-MIR166d | 112.67 | 39.67 | 35.08 | 31.16 | 14.79 | 441.17 | 10.92 |
| gene:ENSRNA049996345 | ppe-MIR172b | U | U | U | 0.37 | U | U | 0.65 |
| gene:ENSRNA049996353 | ppe-MIR160b | 5.22 | 0.48 | 0.50 | 3.30 | 0.07 | 0.55 | 0.60 |
| gene:ENSRNA049996362 | ppe-MIR172d | 1.56 | 0.44 | 15.15 | 0.65 | 0.06 | 1.09 | 10.17 |
| gene:ENSRNA049996366 | ppe-MIR393a | 0.52 | 0.05 | 0.60 | 1.33 | 0.60 | U | 0.56 |
| gene:ENSRNA049996375 | ppe-MIR169f | U | U | 0.10 | U | U | 0.55 | 0.05 |
| gene:ENSRNA049996390 | ppe-MIR393b | U | 0.02 | 4.78 | 0.44 | 1.98 | 75.74 | 0.60 |
| gene:ENSRNA049996398 | ppe-MIR828 | 1.04 | 0.02 | U | U | U | 7.66 | 0.33 |
| gene:ENSRNA049996404 | ppe-MIR171d | U | 0.14 | 3.89 | 2.15 | 0.01 | 1.78 | 0.05 |
| gene:ENSRNA049996411 | ppe-MIR166c | U | 4.87 | 0.40 | 0.34 | 1.14 | 8.07 | U |
| gene:ENSRNA049996417 | ppe-MIR6257 | U | 0.12 | U | U | U | U | 0.09 |
| gene:ENSRNA049996450 | ppe-MIR399c | U | U | U | 0.14 | 0.02 | U | U |
| gene:ENSRNA049996487 | ppe-MIR164d | 0.78 | 0.60 | 1.59 | 0.34 | 0.06 | 0.14 | U |
| gene:ENSRNA049996496 | ppe-MIR8123 | U | U | 0.10 | U | U | U | 0.14 |
| gene:ENSRNA049996508 | ppe-MIR6289 | U | 0.02 | U | U | U | U | U |
| gene:ENSRNA049996514 | ppe-MIR399n | U | U | U | 0.03 | 0.04 | U | U |
| gene:ENSRNA049996520 | ppe-MIR1511 | U | 0.92 | 0.30 | 0.14 | U | 0.68 | 0.05 |
| gene:ENSRNA049996526 | ppe-MIR6286 | U | U | 0.10 | U | U | U | U |
| gene:ENSRNA049996542 | ppe-MIR477b | 0.26 | U | 0.20 | U | U | U | U |
| gene:ENSRNA049996546 | ppe-MIR482c | 655.13 | U | 0.60 | 266.26 | 67.70 | 0.27 | 0.28 |
| gene:ENSRNA049996556 | ppe-MIR156f | U | 0.05 | 67.88 | 2.96 | 0.07 | 4.78 | 17.79 |
| gene:ENSRNA049996563 | ppe-MIR156h | U | 0.85 | 45.95 | U | 0.01 | 5.47 | 2.04 |
| gene:ENSRNA049996584 | ppe-MIR171h | 60.77 | 7.15 | 1.00 | 0.82 | U | 0.14 | 0.05 |
| gene:ENSRNA049996592 | ppe-MIR171a | 168.22 | 0.09 | 18.44 | 0.03 | 0.03 | 1.37 | U |
| gene:ENSRNA049996598 | ppe-MIR171e | 0.52 | 0.21 | 0.40 | U | 0.00 | 0.41 | U |
| gene:ENSRNA049996605 | ppe-MIR169c | U | 0.02 | U | 0.03 | 0.00 | U | U |
| gene:ENSRNA049996610 | ppe-MIR5225 | 0.26 | U | U | 0.44 | 0.02 | U | U |
| gene:ENSRNA049996616 | ppe-MIR3627 | U | U | 0.10 | 0.17 | 0.01 | 0.41 | U |
| gene:ENSRNA049996627 | ppe-MIR156i | 0.26 | 0.37 | U | U | 0.02 | 79.57 | 1.95 |
| gene:ENSRNA049996633 | ppe-MIR171c | 1.04 | 0.23 | U | 0.17 | 0.01 | 0.41 | 0.09 |
| gene:ENSRNA049996642 | ppe-MIR169i | 0.26 | U | U | U | 0.00 | U | U |
| gene:ENSRNA049996647 | ppe-MIR169g | 2.61 | 0.05 | U | U | 0.01 | 0.41 | U |
| gene:ENSRNA049996654 | ppe-MIR397 | 59.72 | 0.07 | U | 0.07 | 0.01 | 0.14 | U |
| gene:ENSRNA049996662 | ppe-MIR399b | U | 0.48 | 0.30 | 7.25 | 1.42 | 5.88 | U |
| gene:ENSRNA049996670 | ppe-MIR399m | U | U | U | 0.03 | 0.01 | U | U |
| gene:ENSRNA049996678 | ppe-MIR2111c | U | U | U | 0.44 | U | U | U |
| gene:ENSRNA049996684 | ppe-MIR2111a | U | U | U | 0.14 | 0.00 | 0.27 | U |
| gene:ENSRNA049996692 | ppe-MIR2111b | U | U | U | 0.03 | 0.03 | U | 0.09 |
| gene:ENSRNA049996698 | ppe-MIR160a | 14.60 | 0.51 | U | 0.48 | U | 0.96 | 0.37 |
| gene:ENSRNA049996707 | ppe-MIR7125 | U | 0.05 | U | 13.90 | 0.15 | U | U |
| gene:ENSRNA049996716 | ppe-MIR6266a | U | U | U | 0.03 | U | U | U |
| gene:ENSRNA049996722 | ppe-MIR6266b | U | U | U | U | U | 0.55 | U |
| gene:ENSRNA049996730 | ppe-MIR6291a | U | U | 0.10 | U | U | U | U |
| gene:ENSRNA049996743 | ppe-MIR6271 | U | 0.02 | 0.10 | U | U | U | U |
| gene:ENSRNA049996749 | ppe-MIR6267a | U | U | U | 1.06 | 0.08 | 0.14 | U |
| gene:ENSRNA049996757 | ppe-MIR169h | U | U | U | U | U | 0.27 | U |
| gene:ENSRNA049996768 | ppe-MIR6292 | U | U | U | U | 0.00 | U | U |
| gene:ENSRNA049996775 | ppe-MIR6267b | U | U | U | U | 0.01 | 0.14 | U |
| gene:ENSRNA049996784 | ppe-MIR6267c | U | 0.02 | U | U | U | 0.14 | U |
| gene:ENSRNA049996792 | ppe-MIR6272 | U | U | U | U | 0.00 | U | U |
| gene:ENSRNA049996796 | ppe-MIR6269 | U | 0.05 | U | U | U | U | U |
| gene:ENSRNA049996803 | ppe-MIR398b | U | 0.16 | 12.56 | 101.05 | U | 0.68 | 0.09 |
| gene:ENSRNA049996813 | ppe-MIR398a | 295.75 | 1.89 | 319.15 | 2.11 | 0.02 | 0.27 | 11.47 |
| gene:ENSRNA049996825 | ppe-MIR6270 | U | 0.09 | 0.10 | U | U | U | U |
| gene:ENSRNA049996833 | ppe-MIR168 | 30.51 | 44.40 | 288.45 | 110.75 | 39.35 | 1084.13 | 32.29 |
| gene:ENSRNA049996854 | ppe-MIR6273 | U | 0.02 | 0.10 | 0.03 | U | 0.14 | U |
| gene:ENSRNA049996862 | ppe-MIR399a | 2.09 | 1.52 | 3.69 | 3.51 | 0.10 | 0.14 | U |
| gene:ENSRNA049996870 | ppe-MIR6275 | U | U | 0.10 | 0.03 | U | U | U |
| gene:ENSRNA049996876 | ppe-MIR319b | U | U | U | U | 0.02 | U | U |
| gene:ENSRNA049996883 | ppe-MIR477 | 0.26 | 0.07 | U | U | U | 15.18 | U |
| gene:ENSRNA049996894 | ppe-MIR166e | 296.01 | 13.45 | 17.94 | 176.35 | 1.45 | 1012.90 | U |
| gene:ENSRNA049996899 | ppe-MIR8130 | U | 0.05 | U | 0.07 | U | U | 0.05 |
| gene:ENSRNA049996904 | ppe-MIR171g | 2.61 | 0.72 | U | 0.07 | U | 2.19 | 0.09 |
| gene:ENSRNA049996910 | ppe-MIR162 | 230.03 | 582.74 | 71.37 | 1697.31 | 300.31 | 0.68 | 213.56 |
| gene:ENSRNA049996921 | ppe-MIR319a | 0.78 | 0.12 | 0.10 | U | 3.15 | 3.55 | 0.46 |
| gene:ENSRNA049996926 | ppe-MIR156g | 0.26 | U | U | 3.34 | 0.01 | 6.97 | 1.81 |
| gene:ENSRNA049996936 | ppe-MIR159 | 2747.28 | 304.59 | 2908.03 | 355.22 | 75.07 | 972.57 | 972.57 |
| gene:ENSRNA049996946 | ppe-MIR858 | 15.13 | 0.78 | 26.11 | 0.17 | 0.00 | 0.27 | U |
| gene:ENSRNA049996956 | ppe-MIR6294 | U | U | U | U | 0.00 | U | U |
| gene:ENSRNA049996963 | ppe-MIR156b | 0.26 | 0.12 | 2.09 | 2.76 | 2.37 | 1621.27 | U |
| gene:ENSRNA049996971 | ppe-MIR390 | 25.30 | 0.30 | 0.40 | 0.17 | U | 11.48 | 1.25 |
| gene:ENSRNA049996977 | ppe-MIR172c | 0.26 | 3.05 | 0.70 | 0.10 | 0.04 | U | 66.39 |
| gene:ENSRNA049996985 | ppe-MIR6288b | U | U | U | U | U | 0.14 | U |
| gene:ENSRNA049996993 | ppe-MIR172a | 0.26 | 1.92 | 16.25 | 0.17 | 0.10 | 0.96 | 0.98 |
| gene:ENSRNA049997001 | ppe-MIR6276 | U | U | U | U | U | 0.14 | U |
| gene:ENSRNA049997007 | ppe-MIR8126 | 0.52 | 0.23 | U | 0.10 | 0.01 | 0.55 | U |
| gene:ENSRNA049997023 | ppe-MIR2111d | U | 0.07 | 0.10 | 0.10 | 0.01 | 0.14 | U |
| gene:ENSRNA049997029 | ppe-MIR164a | 1.83 | 208.48 | 63.69 | 50.78 | 18.43 | 0.82 | 1.02 |
| gene:ENSRNA049997035 | ppe-MIR6295 | U | 0.07 | 0.30 | U | U | U | U |
| gene:ENSRNA049997040 | ppe-MIR164c | 0.52 | 5.26 | 130.57 | 0.54 | 0.01 | U | 0.14 |
| gene:ENSRNA049997046 | ppe-MIR167c | 25.56 | 0.32 | 14.15 | 1.94 | 0.05 | 2.05 | 202.92 |
| gene:ENSRNA049997051 | ppe-MIR6277 | U | U | U | U | U | 0.14 | U |
| gene:ENSRNA049997057 | ppe-MIR6296 | U | U | U | 0.03 | U | 0.27 | U |
| gene:ENSRNA049997062 | ppe-MIR6274b | U | U | 0.10 | U | U | U | U |
| gene:ENSRNA049997066 | ppe-MIR6291b | U | U | U | 0.03 | U | 0.14 | U |
| gene:ENSRNA049997079 | ppe-MIR6280 | U | U | U | U | U | 0.14 | U |
| gene:ENSRNA049997086 | ppe-MIR6291c | U | U | U | 0.14 | U | U | U |
| gene:ENSRNA049997099 | ppe-MIR530 | 24.52 | 0.14 | 1.00 | 0.37 | 0.00 | 10.25 | U |
| gene:ENSRNA049997104 | ppe-MIR6279 | 1.56 | U | U | U | 0.01 | U | U |
| gene:ENSRNA049997115 | ppe-MIR8128 | U | 0.05 | U | U | U | 0.14 | U |
| gene:ENSRNA049997120 | ppe-MIR8124 | U | 0.02 | U | U | U | U | 0.05 |
| gene:ENSRNA049997132 | ppe-MIR156a | U | U | 18.54 | U | U | U | U |
| gene:ENSRNA049997136 | ppe-MIR171b | 0.26 | 0.14 | 10.96 | 0.03 | 0.01 | 2.05 | 0.51 |
| gene:ENSRNA049997141 | ppe-MIR396b | 1.04 | 68.58 | 18.64 | 17.88 | 1.07 | 356.14 | 141.88 |
| gene:ENSRNA049997146 | ppe-MIR156c | U | U | U | 10.86 | 0.10 | 15.86 | U |
| gene:ENSRNA049997151 | ppe-MIR827 | 2.35 | 0.18 | 133.96 | U | 0.00 | 10.25 | 0.37 |
| gene:ENSRNA049997157 | ppe-MIR6281 | U | 0.02 | 0.10 | U | 0.01 | U | U |
| gene:ENSRNA049997163 | ppe-MIR6297b | U | U | 0.10 | 0.03 | U | U | U |
| gene:ENSRNA049997170 | ppe-MIR6297a | U | U | 0.10 | U | 0.00 | U | U |
| gene:ENSRNA049997175 | ppe-MIR482d | U | U | 1.50 | U | U | U | U |
| gene:ENSRNA049997179 | ppe-MIR6282 | U | U | U | 0.54 | 0.01 | U | U |
| gene:ENSRNA049997184 | ppe-MIR6285 | U | U | U | 0.03 | U | U | U |
| gene:ENSRNA049997189 | ppe-MIR535b | 2.09 | U | 4.19 | 6.64 | 1.13 | 1.37 | 8.97 |
| gene:ENSRNA049997194 | ppe-MIR535a | U | U | 0.90 | 2.01 | 0.04 | 0.14 | U |
| gene:ENSRNA049997199 | ppe-MIR6283 | U | U | U | U | 0.00 | U | U |
| gene:ENSRNA049997204 | ppe-MIR166b | 181.00 | 328.11 | 7.87 | 277.40 | 10.78 | 222.70 | 162.92 |
| gene:ENSRNA049997210 | ppe-MIR167b | 635.05 | 1.06 | 1.69 | 7.53 | 0.67 | 5.33 | 22.72 |
| gene:ENSRNA049997214 | ppe-MIR167a | 7.30 | 0.18 | 29.40 | 0.48 | 0.02 | 0.55 | U |
| gene:ENSRNA049997219 | ppe-MIR6284 | U | 0.23 | 0.10 | 1.43 | 0.07 | U | U |
| gene:ENSRNA049997224 | ppe-MIR164b | U | 10.02 | 74.65 | U | 0.15 | U | U |
| gene:ENSRNA050003151 | MIR811 | U | 0.09 | U | U | 0.00 | U | U |
| gene:ENSRNA050003167 | MIR530 | 0.52 | U | 1.89 | 0.14 | 0.37 | U | U |
| gene:ENSRNA050003181 | MIR159 | 151.00 | 0.35 | 3.19 | 2.93 | 0.02 | 13.94 | 0.93 |
| gene:ENSRNA050003184 | MIR169_5 | U | 0.14 | U | 0.75 | 0.01 | 0.14 | U |
| gene:ENSRNA050003201 | MIR169_2 | U | 0.05 | 0.20 | 9.20 | 0.10 | U | 0.09 |
| gene:ENSRNA050003348 | mir-160 | 5.48 | U | 0.70 | U | U | 0.68 | U |
| gene:ENSRNA050003493 | MIR530 | 0.52 | 0.02 | U | 0.10 | 2.46 | U | U |
| gene:ENSRNA050003595 | mir-399 | U | U | U | U | U | U | 0.42 |
| gene:ENSRNA050003605 | MIR159 | U | U | 6.98 | 41.31 | 1.69 | 63.71 | 0.19 |
| gene:ENSRNA050003608 | MIR159 | U | 0.02 | 0.40 | 14.20 | 2.91 | 0.96 | 0.05 |
| gene:ENSRNA050003611 | MIR159 | U | 0.07 | U | 0.07 | 0.01 | U | U |
| gene:ENSRNA050003614 | MIR159 | U | 0.05 | U | 0.03 | U | U | U |
| gene:ENSRNA050003617 | MIR159 | U | 0.02 | 0.20 | U | 0.00 | 0.27 | 0.09 |
| gene:ENSRNA050003691 | MIR169_2 | U | 0.05 | 0.10 | U | 0.00 | U | 0.19 |
| gene:ENSRNA050003695 | MIR398 | U | 0.16 | 10.86 | 10.39 | 0.77 | U | 0.09 |
| gene:ENSRNA050003783 | MIR390 | U | 0.48 | 163.26 | 1.57 | U | 24.06 | U |
| gene:ENSRNA050003799 | MIR159 | 21.65 | 0.09 | 35.58 | U | U | U | U |
| gene:ENSRNA050003808 | mir-156 | 307.22 | 4.82 | 12.66 | 0.03 | U | 3.96 | 15.66 |
| gene:ENSRNA050003821 | MIR396 | 22.17 | 320.45 | 320.45 | 320.45 | 320.45 | 368.71 | 513.63 |
| gene:ENSRNA050003825 | MIR1122 | U | U | U | 0.48 | 0.40 | U | U |
| gene:ENSRNA050004107 | MIR159 | 1.56 | 0.90 | 31.30 | 0.34 | 0.05 | U | 0.05 |
| gene:ENSRNA050004132 | MIR530 | U | U | U | 0.03 | U | U | 0.09 |
| gene:ENSRNA050004138 | mir-399 | U | 0.12 | U | 0.03 | U | U | U |
| gene:ENSRNA050004140 | mir-399 | U | 0.02 | U | U | U | U | U |
| gene:ENSRNA050004158 | mir-399 | U | U | U | 0.03 | 0.04 | U | 0.05 |
| gene:ENSRNA050004180 | MIR169_2 | U | U | U | U | U | 0.27 | U |

Small RNA-sequencing was conducted with total RNA isolated from 0.1 g of fruits and vegetables, and 0.05-0.1 g of fats and oils. Reads were aligned with the Prunus persica genome (annotation NCBIv2.52). Results are presented as normalized reads from a plant product sample. U, undetectable.

**Table S2.** Putative human target genes of plant miR482f predicted with the bioinformatic programs psRNATarget (scoring schemas V1 and V2) and TAPIR.

| **psRNATarget. Scoring Schema V1** | | | | |
| --- | --- | --- | --- | --- |
| **Target accession** | **Expectation** | **UPE** | **mRNA target aligned fragment (5’-3’)** | **Inhibitory effect** |
| **NM_145912\|NFAM1** | 1.5 | 18.312 | 2639-[UGAAUGGGUGGAGGAGGGAAGA]-2660 | Translation |
| NM_152405\|JMY | 2.0 | 13.288 | 4639-[GGAAUUGGUGGA-UAGGAAAGA]-4659 | Translation |
| NM_025142\|TAOK1 | 3.0 | 24.106 | 2350-[UGGAUGGGUGGAAUAGGGAAGC]-2371 | Translation |
| NM_000088\|COL1A1 | 3.0 | 24.795 | 895-[GUGAUGGGUGGGGUGGGGAGGG]-916 | Cleavage |
| **NM_197949\|CLEC7A** | 3.0 | 9.027 | 57-[AGGAAGGGUGGAGAAGGAGAGA]-78 | Translation |
| **NM_022570\|CLEC7A** | 3.0 | 2.735 | 2-[AGGAAGGGUGGAGAAGGAGAGA]-23 | Translation |
| **psRNATarget. Scoring Schema V2** | | | | |
| **Target accession** | **Expectation** | **UPE** | **mRNA target aligned fragment (5’-3’)** | **Inhibitory effect** |
| **NM_145912\|NFAM1** | 2.0 | N/A | 2639-[UGAAUGGGUGGAGGAGGGAAGA]-2660 | Cleavage |
| NM_000088\|COL1A1 | 2.5 | N/A | 895-[GUGAUGGGUGGGGUGGGGAGGG]-916 | Cleavage |
| NM_003718\|CDK13 | 2.5 | N/A | 663-[ACUAUGGGUGGGGUGGGGAGGG]-684 | Cleavage |
| NM_025142\|TAOK1 | 3.0 | N/A | 2350-[UGGAUGGGUGGAAUAGGGAAGC]-2371 | Translation |
| **NM_197949\|CLEC7A** | 3.0 | N/A | 57-[AGGAAGGGUGGAGAAGGAGAGA]-78 | Cleavage |
| **NM_022570\|CLEC7A** | 3.0 | N/A | 2-[AGGAAGGGUGGAGAAGGAGAGA]-23 | Cleavage |
| NM_001170765\|LCOR | 3.5 | N/A | 5122-[GGGGAGGGUGGAGUAGGAUGGA]-5143 | Cleavage |
| NM_001170766\|LCOR | 3.5 | N/A | 2626-[GGGGAGGGUGGAGUAGGAUGGA]-2647 | Cleavage |
| NM_001136265\|IFFO2 | 3.5 | N/A | 3054-[AGAGGGGGUGGGGUGGGGGAGA]-3075 | Cleavage |
| NM_018316\|KLHL26 | 4.0 | N/A | 539-[GGGGUGGGUGGAGGAGGAGGGC]-560 | Cleavage |
| NM_001136022\|NFATC4 | 4.0 | N/A | 969-[GGAGUGUGUGGAGGAGGGAGGA]-990 | Cleavage |
| NM_001198965\|NFATC4 | 4.0 | N/A | 207-[GGAGUGUGUGGAGGAGGGAGGA]-228 | Cleavage |
| NM_152405\|JMY | 4.0 | N/A | 4639-[GGAAUUGGUGGA-UAGGAAAGA]-4659 | Translation |
| NM_198993\|STAC2 | 4.0 | N/A | 216-[GGGAUGGGGUGGGGUGGGAGGGA]-238 | Cleavage |
| NM_177477\|LYNX1 | 4.0 | N/A | 1000-[GGGGAGGGUGGGGUGGGGAGGG]-1021 | Cleavage |
| NM_020709\|PNMAL2 | 4.0 | N/A | 574-[GGAAGGGGAGGAGGAGGAAAGG]-595 | Cleavage |
| NM_145912\|NFAM1 | 4.5 | N/A | 3449-[GGCUUGAGUGGAGUAGGUGAGG]-3470 | Cleavage |
| NM_024007\|EBF1 | 4.5 | N/A | 2380-[GGGGUGGGUGGGGGAGGGGAGU]-2401 | Cleavage |
| NM_021096\|CACNA1I | 4.5 | N/A | 851-[GGGGUGGGUGGAGCAGGAGUGG]-872 | Cleavage |
| NM_031464\|RPS6KL1 | 4.5 | N/A | 1530-[GGAAGGGGUGGGGUCGGGGAGG]-1551 | Cleavage |
| NM_014823\|WNK1 | 4.5 | N/A | 189-[GGAAUGGGAGAGGAAGGAAAGA]-210 | Cleavage |
| NM_012298\|CAND2 | 5.0 | N/A | 569-[GGAAUGGGUUGGGUAGGGAACU]-590 | Cleavage |
| NM_001105568\|KIF13A | 5.0 | N/A | 979-[GGGGUGGGUGGGGUGGGAGGUG]-1000 | Cleavage |
| NM_001198973\|KIAA1522 | 5.0 | N/A | 1696-[GGAAUGGCUGGAGGUGGGAGAGA]-1718 | Cleavage |
| **TAPIR** | | | | |
| **Target accession** | **Score** | **MFE ratio** | **mRNA target aligned fragment (5’-3’)** | |
| **NM_145912\|NFAM1** | 4.0 | 0.79 | 2639-[UGAAUGGGUGGAGGAGGGAAGA]-2660 | |
| NM_170724\|PKHD1 | 3.0 | 0.77 | 320-[GAUAUGGG-GGAGUAGGAAAGA]-341 | |
| NM_001174146\|LMX1B | 4.0 | 0.76 | 4205-[GGACUGGG-GGAGCAGGAAAGA]-4226 | |
| **NM_197949\|CLEC7A** | 4.0 | 0.82 | 58-[GGAA-GGGUGGAGAAGGAGAGA]-79 | |
| **NM_022570\|CLEC7A** | 4.0 | 0.82 | 3-[GGAA-GGGUGGAGAAGGAGAGA]-24 | |

Mature sequence of miR482f (5’-UCUUUCCUACUCCACCCAUUCC-3’) was aligned with the cDNA library “Homo sapiens (human), transcript, Human genomic sequencing project” (available at psRNATarget server). Target accession of transcripts highlighted in bold indicates common putative target genes between psRNATarget schemas V1 and V2 and TAPIR. N/A, non applicable.

|  |
| --- |
| 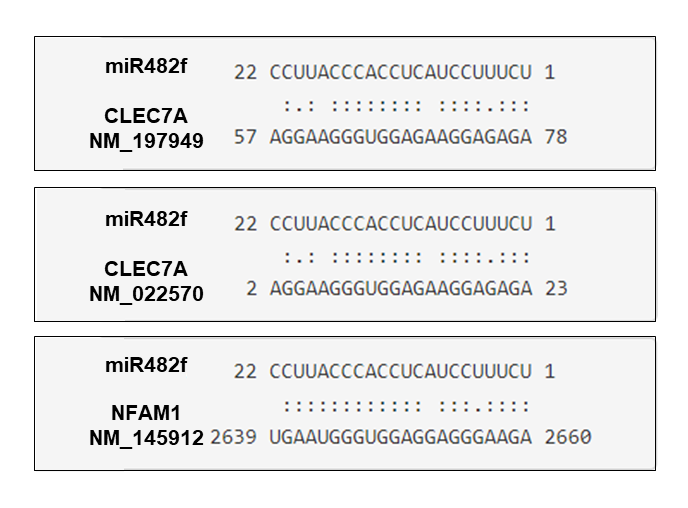 |

**Figure S1.** *In silico* prediction alignment between plant miR482f and the putative target transcripts *CLEC7A* and *NFAM1*. The prediction programs psRNATarget scoring schemas V1 and V2 and TAPIR were applied to predict human target genes of the mature sequence of miR482f (5’-UCUUUCCUACUCCACCCAUUCC-3’).

**Table S3.** *In silico* identification of putative human target genes of plant miR482c-5p predicted with the bioinformatic programs psRNATarget (scoring schemas V1 and V2) and TAPIR.

| **psRNATarget. Scoring Schema V1** | | | | |
| --- | --- | --- | --- | --- |
| **Target accession** | **Expectation** | **UPE** | **mRNA target aligned fragment (5’-3’)** | **Inhibitory effect** |
| **NM_001160332\|NFASC** | 2.5 | 23.579 | 4116-[CAUCACAAACAGCCCAUUUG]-4135 | Cleavage |
| **NM_006068\|TLR6** | 3.0 | 12.595 | 2413-[UAUCCCAAACGGCACAUUCU]-2432 | Cleavage |
| **psRNATarget. Scoring Schema V2** | | | | |
| **Target accession** | **Expectation** | **UPE** | **mRNA target aligned fragment (5’-3’)** | **Inhibitory effect** |
| NM_032420\|PCDH1 | 2.0 | N/A | 45-[GCUCCCAAAUGGCCCAUUCC]-64 | Cleavage |
| **NM_001160332\|NFASC** | 2.5 | N/A | 4116-[CAUCACAAACAGCCCAUUUG]-4135 | Cleavage |
| **NM_006068\|TLR6** | 2.5 | N/A | 2413-[UAUCCCAAACGGCACAUUCU]-2432 | Cleavage |
| NM_001137669\|RGSL1 | 3.0 | N/A | 167-[AAUCUCAUACAGCCCAUUCA]-186 | Cleavage |
| NM_197941\|ADAMTS6 | 3.0 | N/A | 2755-[AUUUCCAAAUAGUCCAUUUC]-2774 | Cleavage |
| NM_001099287\|NIPAL4 | 3.0 | N/A | 619-[AAUUUCAACCAGCUCAUUCC]-638 | Cleavage |
| NM_001243084\|HIF1A | 3.0 | N/A | 189-[GAUCACAGACAGCUCAUUUU]-208 | Cleavage |
| NM_181054\|HIF1A | 3.0 | N/A | 335-[GAUCACAGACAGCUCAUUUU]-354 | Cleavage |
| NM_001098811\|SEPT8 | 3.5 | N/A | 1088-[CAUCCUCAGCAGCUCAUUCG]-1107 | Cleavage |
| NM_004288\|CYTIP | 3.5 | N/A | 174-[CAACCUAAACAGCCAAUUUC]-193 | Cleavage |
| NM_178422\|PAQR7 | 3.5 | N/A | 619-[UGUCCCACACGGCCUGUUCC]-638 | Cleavage |
| NM_002657\|PLAGL2 | 3.5 | N/A | 674-[CAUUCCCAUCAGUCCAUUCC]-693 | Cleavage |
| NM_152734\|C6orf89 | 3.5 | N/A | 4416-[CAUCCCCAGCAGCCUCUUCC]-4435 | Cleavage |
| NM_031455\|CCDC3 | 3.5 | N/A | 932-[GCUCCCAAAGGGCUCAUUCC]-951 | Translation |
| NM_001199140\|AMMECR1L | 3.5 | N/A | 1162-[AUUUCCAAAUAGCUCAUUUU]-1181 | Cleavage |
| NM_001304\|CPD | 3.5 | N/A | 657-[ACUUCUAAAUAGCCCAUUUU]-676 | Cleavage |
| NM_001267594\|SENP1 | 3.5 | N/A | 675-[AGUUCCAAGCAGUCCUUUCC]-694 | Cleavage |
| NM_031453\|FAM107B | 3.5 | N/A | 1769-[GAUCUUGAAAAGCCCAUUUC]-1788 | Translation |
| NM_153836\|CREG2 | 4.0 | N/A | 998-[CAUCCCAGACAGCUCACUGC]-1017 | Cleavage |
| NM_001142497\|MLKL | 4.0 | N/A | 588-[CAGCCCAAAUUGCCCAUUCA]-607 | Translation |
| NM_032679\|ZNF577 | 4.0 | N/A | 56-[UAUCCCAGAGAGCUCAUUUA]-75 | Translation |
| NM_001166245\|HPSE2 | 4.0 | N/A | 1052-[CUUCCCAAGUAGCCUAUUUG]-1071 | Cleavage |
| NM_001166246\|HPSE2 | 4.0 | N/A | 1254-[CUUCCCAAGUAGCCUAUUUG]-1273 | Cleavage |
| NM_153836\|CREG2 | 4.5 | N/A | 2215-[AAUUCCAAAUAAUCUAUUUU]-2234 | Cleavage |
| **TAPIR** | | | | |
| **Target accession** | **Score** | **MFE ratio** | **mRNA target aligned fragment (5’-3’)** | |
| **NM_001160332\|NFASC** | 3.0 | 0.79 | 4116-[CAUCACAAACAGCCCAUUUG]-4135 | |
| **NM_006068\|TLR6** | 4.0 | 0.77 | 2413-[UAUCCCAAACGGCACAUUCU]-2432 | |
| NM_032420\|PCDH1 | 4.0 | 0.77 | 45-[GCUCCCAAAUGGCCCAUUCC]-64 | |
| NM_001137669\|RGSL1 | 3.5 | 0.72 | 167-[AAUCUCAUACAGCCCAUUCA]-187 | |
| NM_017955\|CDCA4 | 4.0 | 0.70 | 121-[AAUCCCAGAACAGCCCAUUAC]-142 | |

Mature sequence of miR482c-5p (5’-GGAAUGGGCUGUUUGGGAUG-3’) was aligned with the cDNA library “Homo sapiens (human), transcript, Human genomic sequencing project” (available at psRNATarget server). Target accession of transcripts highlighted in bold indicates common putative target genes between psRNATarget schemas V1 and V2 and TAPIR. N/A, non applicable.

|  |
| --- |
| 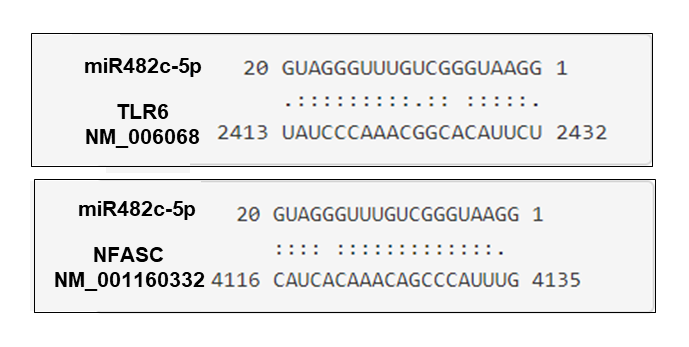 |

**Figure S2.** *In silico* prediction alignment between plant miR482c-5p and the putative target transcripts *TLR6* and *NFASC*. The prediction programs psRNATarget scoring schemas V1 and V2 and TAPIR were applied to predict human target genes of the mature sequence of miR482c-5p (5’-GGAAUGGGCUGUUUGGGAUG-3’).

|  |
| --- |
|  |
| \| (**A**) \| (**B**) \| \| --- \| --- \| |
|  |

**Figure S3.** GeneCodis4 bar chart plots for the Gene Ontology (GO) Biological Processes analyses of (A) *NFAM1* and *CLEC7A* (putative target genes of miR482f), and (B) *NFASC* and *TLR6* (putative target genes of miR482c-5p). Each bar char plot present 20 terms.

|  |  |
| --- | --- |
| (**A**) | (**B**) |

**Figure S4.** Cq values of plant miRNA mimics **(A)** miR482f and **(B)** miR482c-5p after mimic transfection of human THP-1 macrophage-like cells. THP-1 monocytes differentiated to macrophages for 48 h were transfected with 60 nM of miRVana mimics miR482f (5’-UCUUUCCUACUCCACCCAUUCC-3’), miR482c-5p (5’-GGAAUGGGCUGUUUGGGAUG-3’), and a scramble sequence as a negative control (NC). To evaluate miRNA expression, qPCR was performed after 6 h of transfection. Results are the Cq value mean ± standard error of the mean (SEM) (NC, n=2; miR482f, n=3; miR482c-5p, n=3). Abbreviations: NC, Negative Control. Significance refers to the comparison of miR482f or miR482c-5p with respect to the negative control. p-value: *** p<0.001.
